# Cyclopropane-Containing Fatty Acids from the Marine Bacterium *Labrenzia* sp. 011 with Antimicrobial and GPR84 Activity

**DOI:** 10.3390/md16100369

**Published:** 2018-10-08

**Authors:** Jamshid Amiri Moghaddam, Antonio Dávila-Céspedes, Stefan Kehraus, Max Crüsemann, Meryem Köse, Christa E. Müller, Gabriele Maria König

**Affiliations:** 1 Institute for Pharmaceutical Biology, University of Bonn, Nussallee 6, 53115 Bonn, Germany; jamirimoghaddam@uni-bonn.de (J.A.M.); adavila49@gmail.com (A.D.-C.); kehraus@uni-bonn.de (S.K.); mcruesem@uni-bonn.de (M.C.); 2 Pharmaceutical Institute, Pharmaceutical Chemistry I, An der Immenburg 4, D-53121 Bonn, Germany; mkoese@uni-bonn.de (M.K.); christa.mueller@uni-bonn.de (C.E.-M.)

**Keywords:** Roseovarius oyster disease, *Labrenzia*, cyclopropane containing fatty acids, antimicrobials, inflammation, bioinformatics, *Pseudoroseovarius crassostreae*, *Roseovarius crassostreae*, *Aliiroseovarius crassostreae*, GPR84

## Abstract

Bacteria of the family Rhodobacteraceae are widespread in marine environments and known to colonize surfaces, such as those of e.g., oysters and shells. The marine bacterium *Labrenzia* sp. 011 is here investigated and it was found to produce two cyclopropane-containing medium-chain fatty acids (**1**, **2**), which inhibit the growth of a range of bacteria and fungi, most effectively that of a causative agent of Roseovarius oyster disease (ROD), *Pseudoroseovarius crassostreae* DSM 16950. Additionally, compound **2** acts as a potent partial, β-arrestin-biased agonist at the medium-chain fatty acid-activated orphan G-protein coupled receptor GPR84, which is highly expressed on immune cells. The genome of *Labrenzia* sp. 011 was sequenced and bioinformatically compared with those of other *Labrenzia* spp. This analysis revealed several cyclopropane fatty acid synthases (CFAS) conserved in all *Labrenzia* strains analyzed and a putative gene cluster encoding for two distinct CFASs is proposed as the biosynthetic origin of **1** and **2**.

## 1. Introduction

Marine ecosystems host distinct bacterial communities, which cover a wide taxonomic range [1]. These bacteria live in biologically competitive environments and produce fascinating and structurally complex natural products, which were endowed with special biological activities [2,3]. These compounds are known to mediate interactions in the respective ecosystems, such as predator-prey interactions, or the prevention of fouling [4,5,6,7].

Marine bacteria have been found associated with physically unprotected soft-bodied organisms and protect them by the production of bioactive secondary metabolites [3]. Indeed, it has been established that epibiotic or symbiotic bacteria play a major role in keeping their host healthy by the action of their secondary metabolites. A specific example of such a relation is the protective role displayed by *Alteromonas* sp. I-2, which colonizes the embryos of the Caribbean shrimp *Palaemon macrodactylus* [8]. This bacterium synthesizes the antifungal agent 2,3-indolinedione, which prevents infection with the fungus *Lagenidium callinectes*, a frequent crustacean pathogen [8]. In the same manner, embryos of the American lobster *Homarus americanus* were reported to be chemically protected from the same pathogen by the antifungal agent 4-hydroxyphenethyl alcohol, derived from the Gram-negative bacterium SG-76 [9].

Within this context, it is of interest that bacteria of the family Rhodobacteraceae have been found to rapidly colonize surfaces, such as those of oysters and shells. They may produce antibacterial compounds, which in turn shape the microbiome by inhibiting the growth of other bacteria [10,11]. Rhodobacteraceae belong to the phylum Alphaproteobacteria, and are especially widespread and abundant in marine environments [12,13]. *Labrenzia* (previously classified *Stappia*) was established as a new genus of the family Rhodobacteraceae [14]. Species of this genus were suggested to protect mollusks, e.g., *Crassostrea virginica* against the bacterial pathogen *Pseudoroseovarius crassostreae* (also *Aliiroseovarius crassostreae*), a causative agent of roseovarius oyster disease (ROD) [15,16,17]. This pathogen has a negative impact on natural oyster populations and on aquaculture activities, which are related to oysters [18]. It was hypothesized that bacterial metabolites may be involved in the protective role of *Labrenzia* spp. However, secondary metabolites from *Labrenzia* spp. are almost unknown. To date, only a polyketide-derived pederin-analog was discovered from *Labrenzia* sp. PHM005, which showed cytotoxic activity against different cancer cell lines in vitro [19]. The latter, at least, reflects the potential of the *Labrenzia* strains to produce bioactive metabolites.

**Figure 1:**
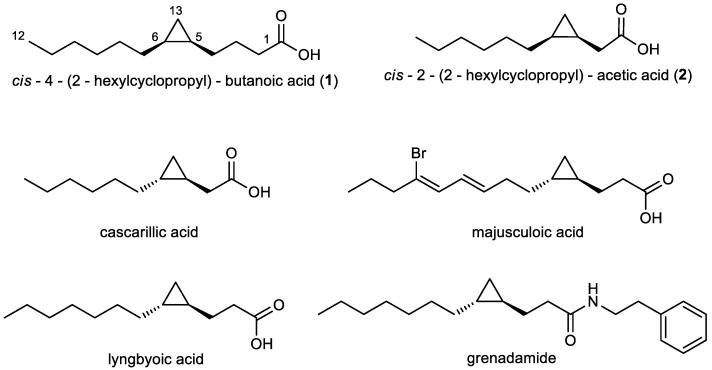
Structures of the cyclopropane fatty acids **1** and **2** (relative configuration) produced by *Labrenzia* sp. strain 011, of cascarillic acid (reproduced from [27], Copyright © 2004 Elsevier Ltd.), lyngbyoic acid (reproduced from [28], Copyright © 2011 The Royal Society of Chemistry), majusculoic acid (reproduced from [23], Copyright © 2005, American Chemical Society), and grenadamide (reproduced from [29], Copyright © 1998, American Chemical Society).

Fatty acids are ubiquitous primary metabolites, however, some of them are of special interest for their antifungal, antibacterial, and antimalarial activity [20]. It was shown that saturated medium-chain fatty acids display effective antifungal activity against a range of plant pathogenic fungi [21]. Antifungal activity was also proven for cyclopropane-containing fatty acids [22]. Marine organisms, in particular, have provided some of the most unusual fatty acids [20]. Majusculoic acid (Figure 1), a brominated cyclopropane fatty acid isolated from marine cyanobacteria, is effective against *Candida albicans* and *C. glabrata* [23].

In the present study we describe the isolation of two medium-chain cyclopropane-containing fatty acids (**1**, **2**) (Figure 1) from the marine bacterium *Labrenzia* sp. strain 011 (Rhodobacteraceae). Beyond the investigation of the chemistry of *Labrenzia*, the aim of our study was to explore the bioactivity and potential biosynthetic pathway of compounds **1** and **2**. Therefore, **1** and **2** were tested against a range of bacteria and fungi including *P. crassostreae*. Moreover, medium-chain fatty acids are considered as the most potent agonists of the orphan G-protein coupled receptor (GPCR) GPR84 [24]. GPR84 is a protein highly expressed on immune cells, e.g., inflammatory macrophages and microglia, and was suggested to be involved in immune defense [25], and may thus be a promising drug target for a variety of inflammatory diseases including Alzheimer’s disease, neuropathic pain, reflux esophagitis, and inflammatory bowel disease [26]. Therefore, compounds **1** and **2** were tested for their ability to interact with GPR84 using cAMP accumulation and β-arrestin recruitment assays. Finally, the genome of *Labrenzia* sp. strain 011 was sequenced and the bioinformatic analysis uncovered different putative biosynthetic genes for cyclopropanation of fatty acids in this marine bacterium.

## 2. Results

### 2.1. Isolation and Structural Elucidation of Compounds ***1*** and ***2***

*Labrenzia* sp. strain 011 was isolated from a marine sample, collected in the coastal area of Kronsgaard, Germany. It was found to be a halophilic bacterium, which exclusively grew under saline conditions. The bacterium was thus cultivated in a marine broth medium supplemented with an adsorber resin. Extraction of the resin with acetone, followed by normal phase fractionation resulted in a fraction with antibacterial activity. Subsequent reversed-phase HPLC separations yielded compounds **1** and **2**.

These compounds are two medium-chain fatty acids, harboring a cyclopropane ring, namely *cis*-4-(2-hexylcyclopropyl)-butanoic acid (**1**), and *cis*-2-(2-hexylcyclopropyl)-acetic acid (**2**) (Figure 1), whereby **1** was identified as a new chemical entity.

The lack of chromophores for **1** and **2** was evident from UV-data(**1**, *λ_max_* = 200 nm; **2**, *λ_max_* = 206 nm). The structures were established by extensive NMR analyses (^1^H, ^13^C, COSY and HMBC, Figures S1–S13, Tables S2 and S3, respectively), and the molecular formulae confirmed by HRESIMS measurements (Figures S14 and S15).

The ^13^C-NMR spectrum of **1** showed 12 resonances between 10 to 35 ppm, attributable to an aliphatic moiety, and an additional resonance at 178.1 ppm, indicating **1** to be a carboxylic acid (Figure S2). From the results of a COSY measurement major fragments of the molecule were deduced. Thus, analysis of the COSY spectrum of **1** gave evidence for connectivities from CH_3_-12 to CH_2_-11, from CH_2_-2 to CH-5 and showed that H-5 also coupled with H_2_-13. The carboxylic group has to be connected to C-2 due to an HMBC correlation from the methylene group CH_2_-2 (δ_H_ 2.40 ppm) to carbon C-1 (δ_C_ 178.1 ppm). Further analysis of the COSY and HMBC spectra showed correlations of the two high-field resonance signals of the magnetically not equivalent H_2_-13 methylene protons H-13a/H-13b (δ_H_ −0.30 ppm, δ_H_ 0.63 ppm) to the methine signal H-6 (δ_H_ 0.68 ppm), and to both C-5 (δ_C_ 15.2 ppm) and C-6 (δ_C_ 15.7 ppm), respectively. This indicated a 1,2-disubstituted cyclopropane ring with three methylene groups placed between the ring and the carboxylic moiety. Four remaining methylene groups, namely H_2_-10 (δ_H_ 1.27 ppm), H_2_-9 (δ_H_ 1.35 ppm), H_2_-8 (δ_H_ 1.25 ppm) and H_2_-7 (δ_H_ 1.35 ppm) were still left and were assigned as shown in Figure 1, evidenced by HMBC correlations from H_3_-12 (δ_H_ 0.88 ppm) to C-10 (δ_C_ 31.9 ppm) and from H_2_-13 (δ_H_ −0.30 ppm, δ_H_ 0.63 ppm) to C-7 (δ_C_ 28.7 ppm), completing the planar structure of compound **1**. The chemical shift of H-13b (δ_H_ −0.30 ppm) suggested the cyclopropane ring to be *cis* configured, confirmed by comparison of ^1^H-NMR chemical shifts of H-5, H-6, and H_2_-13 with structurally related reference compounds [30]. This is to the best of our knowledge, the first description of compound **1** for which we suggest the trivial name labrenzide.

The NMR spectra of compound **2** showed many similarities to those of **1**. The only difference was the absence of two methylene groups as indicated in the dept135 carbon spectrum. HMBC correlations of H_2_-11 (δ_H_ −0.13 ppm, δ_H_ 0.75 ppm) to C-2 (δ_C_ 33.7 ppm), C-3 (δ_C_ 11.1 ppm), and C-4 (δ_C_ 15.5 ppm) and from H_2_-2 (δ_H_ 2.29 ppm, δ_H_ 2.42 ppm) to both C-1 (δ_C_ 180.2 ppm) and C-3 (δ_C_ 11.1 ppm) proved the structure of **2** (Figure 1). The chemical shift of H-11b (δ_H_ −0.13 ppm) suggested the three-membered ring to be *cis* configured [30] as in compound **1**. **2** is thus the *cis* derivative of cascarillic acid (Figure 1) [27].

### 2.2. Antimicrobial Assessment of ***1*** and ***2***

Antimicrobial capacities of **1** and **2** were assessed in disc diffusion tests (DDT) and revealed remarkable growth inhibition for a range of bacterial and fungal organisms, e.g., *Escherichia coli* DSM 498 and *Bacillus megaterium* DSM 32. Due to literature reports [15] relating the presence of *Labrenzia* species to healthy oysters, we also tested the oyster pathogen *P. crassostreae* DSM 16950^T^. Both compounds showed strong activity against *P. crassostreae* close to that of the positive control streptomycin (Table 1). Finally, compound **2** was tested against multidrug-resistant human pathogens, namely *E. coli* I-11276b, Methicillin-resistant *Staphylococcus aureus* (MRSA) LT-1338 and MRSA LT-1334, and was found to also be active (Table 1).

### 2.3. Effects of Compounds ***1*** and ***2*** on the Orphan G-Protein Coupled Receptor GPR84

The GPCR GPR84 is a pro-inflammatory receptor [31,32,33,34], activated by medium-chain fatty acids with a length of 9–14 carbons [16,19]. Since **1** and **2** are analogs of such fatty acids, we anticipated that they may interact with this receptor. Therefore, the isolated fatty acid derivatives **1** and **2**, and the corresponding fatty acids with the same chain length, i.e., decanoic and dodecanoic acid, were investigated in cAMP accumulation, and in β-arrestin recruitment assays (Table 2).

cAMP accumulation assays determined the potency of the test compounds to inhibit forskolin-induced cAMP accumulation in Chinese hamster ovary (CHO) cells, stably expressing human GPR84, whereas the β-arrestin recruitment assays used the enzyme complementation technology.

Compound **1** was inactive in both assays. Compound **2** did neither induce nor block Gi-mediated signaling, however, it showed a concentration-dependent activation of GPR84-dependent β-arrestin recruitment with an EC_50_ value of 114 nM, thus being 53-fold more potent than decanoic acid (EC_50_ = 6.08 μM), and 25-fold more potent than dodecanoic acid (EC_50_ = 2.84 μM) in this assay. The positive controls were both standard agonists and may represent the physiological agonists of the receptor (Table 2, Figure S16). The efficacy was determined to be 54% compared to the full agonist embelin (Table 2). Decanoic and dodecanoic acid displayed an efficacy of 92% and 95%, respectively, under the same conditions. This indicates that compound **2** is a partial agonist of GPR84, and is biased towards β-arrestin recruitment. It does, however not induce Gi-protein coupled signaling.

### 2.4. Bioinformatic Analysis of the Genome of Labrenzia sp. 011 for the Identification of the Putative Biosynthetic Genes of ***1*** and ***2***

In order to elucidate the putative biosynthesis of compounds **1** and **2**, the genome of *Labrenzia* sp. 011 has recently been sequenced (accession number: QCYM00000000) [35]. The resulting genome was compared with further available genome sequences of the genus *Labrenzia*. It was found that all *Labrenzia* strains share between 71.1–99.9% identities at the amino acid level (Figure S17), and orthologous genes in these strains might perform the same functional roles. In a next step, the genomes of *Labrenzia* strains were screened in silico to identify the presence of genes or biosynthetic gene clusters (BGCs) coding for cyclopropane fatty acid synthases (CFAS), which are reported to catalyze the cyclopropanation of unsaturated lipids in bacteria [36,37].

**Figure 2:**
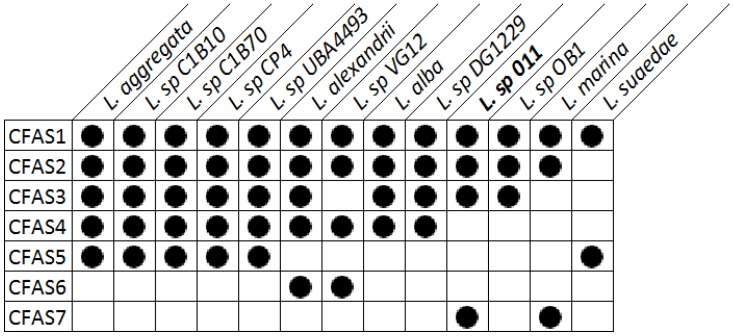
Presence of different types of CFAS genes in the genomes of *Labrenzia* strains, as determined from EDGAR genome comparison.

Regions with conserved CFAS genes in *Labrenzia* strains were aligned. All investigated *Labrenzia* strains harbor between 2–5 different types of CFAS genes in their genomes, some of them highly conserved (Figure 2). *Labrenzia* sp. 011 possesses four different types of CFAS genes, assigned as CFAS1–3 and CAFS7. The first three, i.e., CFAS1–3 are conserved among most *Labrenzia* strains analyzed, while CFAS7 was only present in *Labrenzia* sp. 011 and an *L. marina* strains.

**Figure 3:**
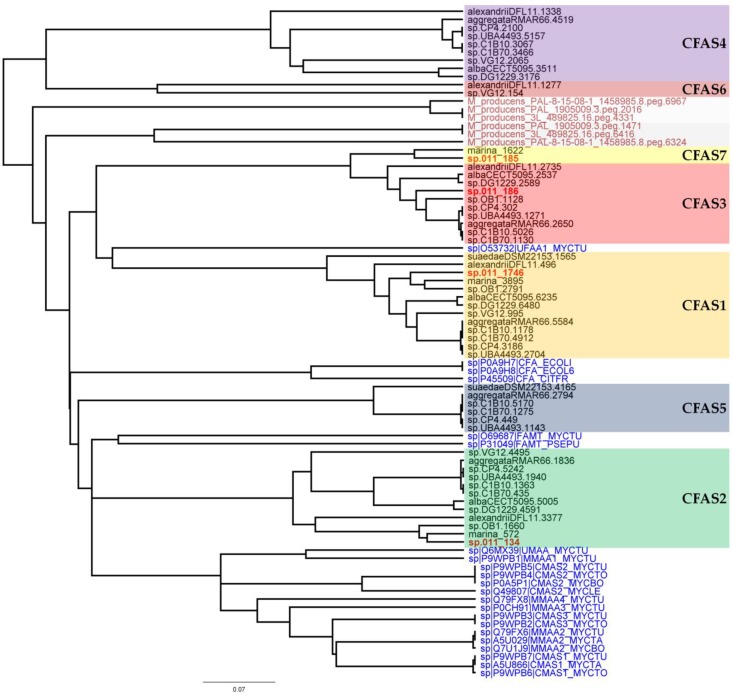
Phylogeny tree of different clades of CFAS genes detected in *Labrenzia* strains based on amino acid sequences. CFAS clades from *Labrenzia* spp. are located in colored boxes. Text in red indicates the CFAS genes from *Labrenzia* sp. 011 and black indicates the annotated CFAS genes from other *Labrenzia* strains. Text in blue indicates the CFAS genes obtained from the UniProtKB protein database with respective codes. Dark red indicates CFAS genes from *Moorea producens* strains namely 3L, PAL, and PAL-8-15-08-1.

In a next step, phylogenetic analyses of the CFAS genes were performed (Figure 3). These included all CFAS genes detected in *Labrenzia* strains, and all described CFAS genes from the UniProtKB protein database, as well as CFAS genes from the marine cyanobacterium *Moorea producens* (formerly classified as *Lyngbya majuscula*) [38]. *M. producens* was earlier reported to produce similar medium-chain length fatty acids with a cyclopropane ring (see lyngbyoic acid in Figure 1) as in **1** and **2** [23].

**Figure 4:**
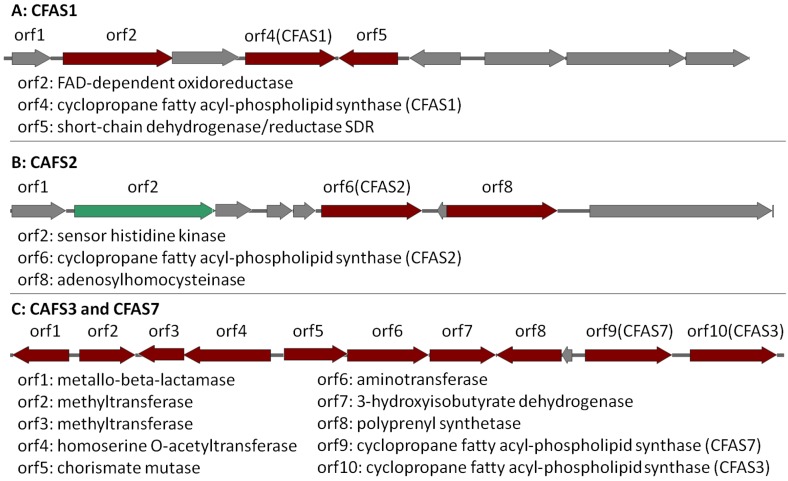
CAFS1, CFAS2 and CFAS3/7 gene clusters in *Labrenzia* sp. 011 assigned as putative BGCs by AntiSMASH (Version 4, Wageningen University, Wageningen, Netherlands). Dark red indicates the biosynthetic genes. Green indicates regulatory genes and grey indicates other genes. Locus accession numbers: QCYM01000001 (CFAS1), QCYM01000010 (CFAS2), and QCYM01000011 (CFAS3/7).

CFAS1 is a conserved gene in all the investigated *Labrenzia* strains (Figure 4A and Figure S18) (75–91% protein identity) with sequences for a short-chain dehydrogenase/reductase (SDR) and a FAD-dependent oxidoreductase in its vicinity (Figure 4A). The closest described CFAS to the CFAS1 clade of *Labrenzia* spp. was *ufaA1* from *Mycobacterium tuberculosis* (strain ATCC 25618) (Figure 3). The enzyme encoded by the latter catalyzes the transfer of a methyl group from S-adenosyl-l-methionine (SAM) to the double bond of oleic acid to produce tuberculostearic acid (syn. 10-methylstearic-acid) [39].

CFAS2 is also conserved (72–90% protein identity) among all *Labrenzia* strains (Figure 4B and Figure S19), except *L. suaedae*, with sequences encoding an adenosylhomocysteinase in its vicinity, which converts S-adenosylhomocysteine to homocysteine and adenosine in methionine biosynthesis. The closest described CFAS to the CFAS2 clade in *Labrenzia* spp. is the methyltransferase of *M. tuberculosis* (Figure 3) which catalyzes the cyclopropanation of the double bonds in mycolates by the addition of a methyl group derived from SAM [40].

Additionally, the CFAS3 gene is conserved in most *Labrenzia* strains (84–92% protein identity), and clustered with a unique CFAS7, which is detected only in *Labrenzia* sp. 011 and *L. marina* (Figure 4C and Figure S20). This BGC also includes sequences for a conserved homoserine *O*-acetyltransferase and a methyltransferase (*MetW*). These might participate in the metabolism of methionine and subsequently provide SAM by using a well-conserved S-adenosylmethionine synthetase present in all the *Labrenzia* strains.

CFAS7 in *Labrenzia* sp. 011 is a distinct CFAS, albeit related to the CFAS3 clade (Figure 3). To date, no other CFASs related to the CAFS3 clade have been reported. *M. producens* produces similar cyclopropane-containing fatty acids, i.e., lyngbyoic acid with a 12-membered carbon chain as **1**, and majusculoic acid with a chain of 14 carbons (Figure 1) [23,28]. Indeed the genomes of *M. producens* strains harbor two distinct clades of CFAS genes (Figure 3). Therefore, the CFAS3 and CFAS7 gene clusters might be responsible for biosynthesis of **1** and **2** in *Labrenzia* sp. 011.

## 3. Discussion

Fatty acids from marine organisms often display unusual structural features, which originate from special biosynthetic pathways [20]. Specifically, modified fatty acids such as cyclopropane fatty acids with antifungal properties were isolated from marine organisms in the past [23]. Their antimicrobial activities have been attributed to their potential to inhibit the synthesis of structural fatty acids in microorganisms or to generate instability in cell membranes [20,22]. A specific mechanism of action for cyclopropyl fatty acids was provided by Kwan et al. [28]. They showed that the cyanobacteria-derived lyngbyoic acid (analog to **1** and **2**) is an efficient inhibitor of the quorum sensing mechanism (QS) of *Pseudomonas aeruginosa*. QS can regulate several behaviors in bacteria, such as secretion of virulence factors, biofilm formation, competence and bioluminescence [41]. The cyclopropane ring is necessary for the QS-inhibitory function of lyngbyoic acid, this way avoiding metabolism *via* β-oxidation [28].

Species of the genus *Labrenzia* were suggested to be protective agents for the oyster *C. virginica* against the halophilic bacterium *P. crassostreae*, a causative agent of ROD [42]. In addition, it was observed that isolates of the genus *Labrenzia*, producing antimicrobially active compounds, were associated with soft corals and the marine sponge *Erylus discophorus* [43,44]. In the present work, compounds **1** and **2** produced by *Labrenzia* sp. strain 011 were found to be active against a range of Gram-negative and Gram-positive bacteria, including multidrug-resistant pathogens and the *P. crassostreae* strain DSM 16950. Compounds **1** and **2** revealed remarkable growth inhibition against the latter pathogen in DDTs (Table 1). This efficacy is comparable to that of the positive control streptomycin. 

Cyclopropane fatty acids also possess antifungal activity. They either inhibit key enzymes responsible for the biosynthesis of fungal fatty acids or cause serious disruptions in fungal membranes [20]. Here, we observed remarkable antifungal activity of **1** and **2** against fungal pathogens. Majusculoic acid from marine cyanobacteria [23] and a C17:0 cyclopropane fatty acid from a *Pseudomonas* spp. [45] are examples of natural cyclopropane fatty acids with proven antifungal activity. Although the precise mechanisms of the antifungal activity of these fatty acids are unknown, certain cyclopropane fatty acids can inhibit fatty acid desaturases [22]. Moreover, medium-chain fatty acids (C8:0–C10:0) showed fungicidal activity against a range of plant pathogenic fungi, whereas long-chain fatty acids such as oleic acid (C18:1) and erucic acid (C22:1) had no inhibitory effect on fungal strains [21]. Fungicidal fatty acids have also been found to increase membrane fluidity and therefore to disrupt functions of the fungal cytoplasmic membrane by inducing the release of intracellular electrolytes and proteins [20]. It was shown that cyclopropane fatty acids disrupt lipid packing and increase the lipid lateral diffusion, resulting in enhanced fluidity [46]. In our case, compound **2** was active against all three fungal test strains, while compound **1** was devoid of activity toward *Eurotium rubrum* DSM 62631 and showed, as compared to **2**, less activity against other fungal strains. The variation in fungicidal activity of **1** and **2** indicates that the inhibitory effects depend on structural motives of the compounds.

Compounds **1** and **2** were also explored concerning their ability to influence GPR84, similar to their potent fatty acid counterparts with the same chain length. GPR84 is an integral membrane protein, which may regulate inflammatory responses. It is suggested that GPR84 can be activated by medium-chain fatty acids, while short- and long-chain saturated and unsaturated fatty acids are inactive [32]. Compounds **1** and **2** are structural analogs of the GPR84 agonists dodecanoic and decanoic acid, respectively. Surprisingly, compound **2**, the decanoic acid analog, proved to be a partial agonist of GPR84 biased towards β-arrestin recruitment. Through this mechanism, compound **2** may inhibit inflammatory effects. In this respect, it is worth noting that cascarillic acid, the *trans* isomer of compound **2** (Figure 1), is the main component of cascarilla essential oil. Interestingly, the latter has been utilized for many years as an inhalant to palliate the inflammatory symptoms of respiratory ailments in folk medicine [27,47,48]. NMR analysis also showed the presence of a small amount of the cis-disubstituted cyclopropane in natural cascarillic acid [27]. However, deeper investigation, including in vivo testing is required in order to assign a role to compound **2** in inflammation.

The biosynthesis of cyclopropane fatty acids uses unsaturated fatty acids as a substrate [37]. Their modification, catalyzed by the CFAS enzymes, occurs in many bacteria and is recognized to play an important role in the adaptation of bacteria to drastic environmental perturbation such as acid or freeze-drying stress [49,50]. The cyclopropanation reaction of unsaturated lipids is well described for long chain fatty acids of *M. tuberculosis* and *E. coli*, and mainly associated with bacterial membranes [37,51]. In general, bacterial CFASs catalyze the transfer of a methyl group from SAM to an inactivated double bond of a lipid chain, followed by deprotonation of the newly attached methyl group and ring closure to form a cyclopropane ring [37]. CFAS genes have highly conserved amino acid sequences and several clades of functional CFAS genes have been identified by phylogenetic analysis [52,53]. Species of genus *Labrenzia* possess several different CFAS genes in their genomes, which indicates their ability to biosynthesize different cyclopropane fatty acids [14]. 

In *Labrenzia* sp. 011 four different CFAS clades were identified and a phylogenetic analysis revealed their relationship with known functional CFAS genes. This analysis grouped the CFAS1 gene with UfaA1 from *M. tuberculosis* H_37_Rv, which synthesizes 19:0Me10 from 18:1ω9 and NADPH [39]. Heterologous expression of UfaA1 revealed this enzyme to be co-located with an FAD-dependent oxidoreductase, which contributes to the reduction reaction that is required for the conversion of 10-methylene octadecanoic acid to 19:0Me10 [39]. Interestingly, in the CFAS1 clade, which is the closest clade to *ufaA1*, the orf2 in the vicinity of CFAS1 is also an FAD-dependent oxidoreductase. Therefore, members of this CFAS1 clade probably perform the same function in *Labrenzia* strains. The fatty acids 11-methyl 18:ω6t and 11-methyl 20:ω6t were indeed isolated from different *Labrenzia* strains [14].

On the other hand, the CFAS2 clade was grouped with SAM-dependent methyltransferases of *Mycobacterium* strains, which are involved in the mycolic acid biosynthesis pathway [53]. Mycolic acids are long-chain fatty acids containing several cyclopropane rings and found in the hydrophobic cell walls of mycobacteria. Functional cyclopropane groups are introduced to the mycolate chain by numerous cyclopropane synthases [53]. The fatty acids cyclo 21:0 and cyclo 19:0 were formerly isolated from *L. alexandrii* and *L. marina* [14]. Therefore, production of such long chain cyclopropane fatty acids is probably related to the CFAS2 clade in *Labrenzia* strains.

To the best of our knowledge, there is no report of CFASs, which catalyze the cyclopropanation of medium-chain fatty acids such as **1** and **2** in bacteria. Therefore, the CFAS3 and CFAS7 in *Labrenzia* sp. 011 are probably responsible for the biosynthesis of these molecules. Additionally, the presence of aminotransferase and chorismate mutase genes in the respective CFAS gene cluster (Figure 4C) is probably related to hitherto unknown compounds with a structure supposedly similar to that of grenadamide (Figure 1) from *M. producens* [28,29], i.e., the cyclopropane containing fatty acids **1** and **2** may also occur as amides. However, experimental studies such as knock-outs of the potential biosynthetic genes have to be performed in order to confirm the biosynthetic origin of compounds **1** and **2** and the presence of further derivatives of the latter.

In conclusion, the marine bacterium *Labrenzia* sp. strain 011 produces two antimicrobial cyclopropane fatty acids (**1** and **2**). Growth inhibition of the pathogenic bacterium *P. crassostreae*, a causative agent of ROD, points toward an ecological relevance of **1** and **2**, however, further experiments have to verify this. The partial agonistic and biased β-arrestin recruitment activity of compound **2** at GPR84 is of pharmacological interest, due to the possible involvement of this receptor in neuroinflammatory processes. In this regard, the search for GPR84 ligands deserves particular attention, in order to elucidate the function of GPR84, which is still considered an orphan receptor. Our genomic analysis revealed several conserved CFAS genes in the genus *Labrenzia*, putatively responsible for methylation and cyclopropanation of long-chain fatty acids. In addition, a gene cluster containing two CFAS genes in the genome of *Labrenzia* sp. 011 is proposed for the biosynthesis of **1** and **2**.

## 4. Experimental Section

### 4.1. General Procedures

UV spectra were recorded on a Perkin-Elmer Lambda 40 with UV WinLab Version 2.80.03 software (Perkin-Elmer, Waltham, MA, USA); quartz cells of 1 cm length were selected. NMR spectra were recorded at a 300 MHz Bruker Avance DPX UltraShield spectrometer (Bruker, Karlsruhe, Germany) in CDCl_3_ as solvent and internal standard; spectra were referenced to residual solvent signals with resonances at δ_H/C_ = 7.26/77.0 ppm. Flash chromatography was conducted on a Reveleris^®^ X2 chromatography system (Büchi, Flawil, Switzerland) with evaporative light scattering detector (ELSD), UV detector, equipped with dry air supply. High-performance liquid chromatography was performed on a Merck-Hitachi HPLC system (Merck-Hitachi, Darmstadt, Germany) equipped with an in-line degasser, L6200A intelligent pump, D-6000 interface, and L-4500 photodiode-array detector. Mass spectra were recorded on a microTOF-Q mass spectrometer (Bruker, Billerica, MA, USA) with ESI-source coupled with an HPLC Dionex Ultimate 3000 (Thermo Scientific, Waltham, MA, USA) using an Agilent Zorbax Eclipse Plus (Agilent, Santa Clara, CA, USA) C_18_ column (2.1 × 50 mm, 1.8 mm I.D.). The column temperature was 45 °C. HPLC begins with 90% H_2_O containing 0.1% acetic acid. The gradient starts after 1 min to 95% acetonitrile (0.1% acetic acid) in 4 min. 2 μL of a 1 mg/mL sample solution was injected to a flow rate of 0.8 mL/min.

### 4.2. Isolation and Taxonomy of the Bacterial Strain

A sample of marine sediment was collected in August 2012 in the coastal area of Kronsgaard, Germany. The sample was air-dried for 24 h and portions thereof were placed on artificial seawater-cycloheximide (ASW-WCX) agar plates. After 5 days of incubation at 30 °C, creamy yellowish colonies were transferred to Petri dishes containing marine agar (Difco 2216) for purification through several passages until an axenic culture was obtained (Figure S21). Based on 16S rDNA (Table S1) alignments, the isolate shares 99% identity with its closest relatives, namely *Labrenzia alba* strain 5OM6 (AN: NR_042378.1) and *L. aggregata* strain RMAR6-6 (AN: CP019630.1). The bacterium was thus identified as *Labrenzia* sp. strain 011. The strain *Labrenzia* sp. 011 is stored in the German Collection of Microorganisms and Cell Cultures (DSMZ) with the accession number DSM 107099.

### 4.3. Cultivation and Isolation Protocol

Cultivation was performed repeatedly in Erlenmeyer flasks (8 flasks, each having a volume of 5 L), each containing 1.5 L of marine broth medium (Difco 2216) and 20 g∙L^−1^ of adsorber resin Sepabeads SP207 (Supelco, St. Louis, MO, USA). Prior to sterilization, pH value of the medium was adjusted to 7.5. The Erlenmeyer flasks were each inoculated with 100 mL of pre-culture in the same medium. The cultures were shaken on a rotary shaker at 120 rpm for 10 days at 30 °C. Bacterial pellet and adsorber resin were separated from the medium by using a filter (pore size 2) and extracted with acetone (8 × 200 mL). After the organic solvent was removed in vacuo, the residue (2.9 g) was dissolved in aqueous methanol (60%) and extracted three times with dichloromethane. The organic phases were mixed and evaporated under vacuum and a lipophilic crude extract (620 mg) was obtained. The extract was further separated by flash chromatography. The fraction of interest (21.2 mg) was obtained by a linear gradient (0–3 min acetone/petroleum ether 0:100, 3–9 min up to 25% acetone, 9–10 min up to 100% acetone, ≈ 20 min 100% acetone at a constant flow rate of 30 mL/min), using a Reveleris silica column (Büchi, Flawil, Switzerland) of 12 g (12 mg–2.4 g sample load capacity, 12 μm). This fraction was collected between min 5 and 6, evaporated under vacuum and dissolved in acetone. The fraction was further fractionated by semi-preparative HPLC using a linear gradient (0–5 min acetonitrile/water 50:50, 5–20 min up to 100% acetonitrile, ≈50 min 100% acetonitrile, containing 0.01 M H_3_PO_4_ each solvent at a constant flow rate of 1 mL/min). An YMC-triart C_18_ column (YMC, Kyoto, Japan, 250 × 4.6 mm I.D., 5 μm) was employed for this step. Two oily colorless substances, namely **1** (1 mg) and **2** (6 mg) were obtained. Their retention time was respectively 22 min (100% acetonitrile) and 17 min (90% acetonitrile).

***cis*-4-(2-hexylcyclopropyl)-butanoic acid 1**: colorless oil; ${\left[\alpha \right]}_{D}^{20}$ -15 (c 0.1, CHCl_3_); UV (acetonitrile) *λ_max_* (ε) 207 nm (2366); ^1^H- and ^13^C-NMR (Table S2); (-) HRESIMS *m*/*z* 211.1677 [M − H]^−^ (calculated for C_13_H_23_O_2_, 211.1698) (Figure S14).

***cis*-2-(2-hexylcyclopropyl)-acetic acid 2**: colorless oil; ${\left[\alpha \right]}_{D}^{20}$ -1 (c 0.1, CHCl_3_); UV (acetonitrile) *λ_max_* (ε) 200 nm (1071); ^1^H- and ^13^C-NMR (Table S3); (-) HRESIMS *m*/*z* 183.1403 [M − H]^−^ (calculated for C_11_H_19_O_2_, 183.1385) (Figure S15).

### 4.4. Genome Sequencing and Assembly

The genomic DNA of *Labrenzia* sp. 011 was isolated as described before [54]. In brief, cell pellets from the one week culture in marine broth liquid medium (see cultivation for details), were harvested. DNA was isolated using the GenElute™ Bacterial Genomic DNA Kit (Sigma-Aldrich, St. Louis, MO, USA). Illumina shotgun paired-end sequencing libraries were generated and sequenced on a MiSeq instrument (Illumina, San Diego, CA, USA). Paired-end reads were combined using the SPAdes assembler v3.10, yielding initial sequence scaffolds [55]. After filtering scaffolds smaller than 1 kb, the remaining scaffolds were determined with QAST (Version 4, Algorithmic Biology Lab, St. Petersburg, Russia) [56]. Genome completeness was estimated using CheckM (Version 1.0.12, University of Queensland, St. Lucia, Australia) [57], using genus level marker genes, and yielded 83.2%. The resulting genome has been deposited at DDBJ/ENA/GenBank under the accession number QCYM00000000. The available genome sequences of different *Labrenzia* strains were obtained from NCBI GenBank: *L. alexandrii* DFL-11: ACCU00000000, *L. aggregata* strain RMAR6-6 chromosome: CP019630, *L. suaedae* strain DSM 22153: FRBW00000000, *L. alba* strain CECT 5095: CXWE00000000, *Labrenzia* sp. CP4: CP011927, *Labrenzia* sp. VG12: CP022529, *Labrenzia* sp. DG1229: AYYG00000000, *Labrenzia* sp. C1B10: AXBY00000000, *Labrenzia* sp. C1B70: AXCE00000000, *Labrenzia* sp. OB1: JSEP00000000, *L. marina* DSM 17023: PPCN00000000, and *Labrenzia* sp. UBA4493: DGNL00000000. GenBank accession numbers of *M. producens* strains: PAL: MKZR00000000.1, PAL-8-15-08-1: CP017599.1, and 3L: AEPQ00000000.1.

### 4.5. Prediction of CFAS Coding Regions

Coding sequences of all analyzed genomes were determined using the RAST (Version 2, Argonne National Laboratory, Lemont, IL, USA) prokaryotic genome annotation server [58]. Therefore, the genetic codes 11 used by most bacteria were applied in classic RAST and the options “automatically fix errors”, “fix frame shifts”, “build metabolic model” and “backfill gaps” were selected.

Biosynthetic gene clusters (BGCs) for specialized metabolites were identified using AntiSMASH v4 (Wageningen University, Wageningen, Netherlands) [59], default parameters and incorporation of the ClusterFinder algorithm were applied. Further, coding regions for cyclopropane fatty acyl-phospholipid synthase (CFAS) were detected using RAST genome browser [58]. 

### 4.6. Genome Comparison and Phylogeny of CFAS

The EDGAR 2.2 genomic pipeline (Bioinformatics & Systems Biology, Giessen, Germany) was used for genome comparison [60]. Therefore, the RAST-annotated GenBank files were uploaded to EDGAR and the core genome, orthologous genes and singletons were identified. The average amino acid identity (AAI) matrix of all conserved genes in the core genome was computed by the BLAST algorithm and visualized as heat map (Figure S17) [60]. The fractions of the genomes with conserved CFAS genes were searched using EDGAR regional alignment to enable comparison of the similar gene regions [60] and mapped using MultiGeneBlast [61]. Multiple sequence alignment and phylogenetic analysis of the CFAS genes were done using Clustal Omega program located in the UniProt database [62].

### 4.7. Antimicrobial Assays

Compounds **1** and **2** were tested against a range of microorganisms, namely the Gram-positive bacterium *Bacillus megaterium* DSM 32, the Gram-negative bacterium *Escherichia coli* JM 83 and *Pseudoroseovarius crassostreae* DSM 16950^T^; the fungal organisms utilized were *Eurotium rubrum* DSM 62631, *Microbotryum violaceum* MB#110229, and *Mycotypha microspora* MB#271115. Tests were performed according to Reference [63]. 50 μg of pure compounds or positive controls were taken from a solution of 1 mg/mL and applied on sterile paper discs on agar plates of the corresponding medium for each test organism. Multidrug-resistant pathogens *Escherichia coli* I-11276b, MRSA LT-1338, MRSA LT-1334 were tested with 4 μg of compound **2** in each disc. Thereafter, suspensions of the organisms were sprayed on the agar and incubated. Miconazole and streptomycin were used as positive controls. Inhibition zones were measured from the edge of the disc to the end of the area free of growth. Only total inhibition (>1 mm) was considered as a positive result.

### 4.8. Biological Assays at the GPR84 Receptor

The recombinant CHO cell line expressing the human GPR84 (CHO-hGPR84) with a β-galactosidase fragment and β-arrestin containing the complementary fragment of the enzyme was purchased from DiscoverX (Fremont, CA, USA). This cell line was used for the β-arrestin recruitment assays as well as for cAMP accumulation assays. The CHO-hGPR84 cells were cultured in F12 medium supplemented with 10% FCS, 100 units/mL penicillin G, 100 μg/mL streptomycin, 800 μg/mL G 418, 300 μg/mL hygromycin B, and 1% ultraglutamin (Invitrogen, Carlsbad, CA or Sigma-Aldrich, St. Louis, MO, USA). Stock solutions of compounds were prepared in DMSO (final DMSO concentration: 1%). Data were analyzed using Graph Pad Prism version 6.0 (San Diego, CA, USA). Concentration-response data were fitted by nonlinear regression to estimate EC_50_ values. 

### 4.9. cAMP Accumulation Assays

cAMP assays were performed as previously described [24]. CHO cells overexpressing the human GPR84 were stimulated with forskolin (10 μM) in the absence (control) or presence of test compound for 15 min. The reaction was stopped by the addition of hot (90 °C) lysis solution containing 4 mM EDTA and 0.01% Triton X-100 in water. cAMP levels were quantified by a radioactive assay using [³H]cAMP (Perkin-Elmer, Rodgau, Germany). The forskolin-induced change in cAMP concentration in the presence of the test compound was expressed as a percentage of the response to forskolin in the absence of agonist (% of control). Three independent experiments, each in duplicates were performed.

### 4.10. β-Arrestin Recruitment Assays

β-arrestin assays were performed as previously described [24]. Briefly, CHO cells expressing the human GPR84 with a β-galactosidase fragment and β-arrestin containing the complementary fragment of the enzyme were incubated with a series of compound dilutions (in DMSO, final DMSO concentration: 1%) for 90 min before adding the detection reagent (DiscoverX^®^, Fremont, CA, USA). After 60 min of incubation at room temperature the luminescence was measured using an NXT plate reader (Perkin-Elmer, Rodgau, Germany). Three to five independent experiments were performed, each in duplicates.

## Figures and Tables

**Table 1:** Antimicrobial activities of compounds **1** and **2** determined in disc diffusion assays.

Organism	Antimicrobial Activity		
**Bacteria**	**Compound 1**	**Compound 2**	**Streptomycin (+)**
*Escherichia coli* DSM 498	2	3	6
*Bacillus megaterium* DSM 32	NA	10	9
*Pseudoroseovarius crassostreae* DSM 16950^T^	5	5	6
*Escherichia coli I-11276b	NT	2	NT
*MRSA LT-1338	NT	2	NT
*MRSA LT-1334	NT	3	NT
**Fungi**	**Compound 1**	**Compound 2**	**Miconazole (+)**
*Eurotium rubrum* DSM 62631	NA	10	8
*Mycotypha microspora* MB#271115	3	6	9
*Microbotryum violaceum* MB#110229	8	10	6

Values are presented as growth inhibition zones in mm produced by 50 μg of each compound or positive control (+) used in disc diffusion tests. *Multidrug-resistant pathogens (MDRP). The amount of compound **2** used against MDRP was 4 μg on each disc. MRSA: Methicillin-resistant *Staphylococcus aureus*. NA: no activity detected; NT: not tested.

**Table 2:** Activities of fatty acid derivatives **1** and **2** at the human GPR84.

Compound	cAMP Assay ^a^		β-Arrestin Assay	
	EC_50_ ± SD (μM) (Receptor Activation) ^b^ *n* = 3	Efficacy ^c^	EC_50_ ± SD (μM) (Receptor Activation) ^d^ *n* = 5	Efficacy ^e^
Decanoic acid	7.42 ± 0.80	100% [26]	6.08 ± 0.63	92% [26]
Dodecanoic acid	8.87 ± 4.46	94%	2.84 ± 0.54	95%
1	>>100 (0%) ^b^	-	>30 (17%) ^d^	-
2	>100 (24%) ^b^	-	0.114 ± 0.135	54%

^a^ Inhibition of forskolin (10 μM) induced increase in cAMP accumulation; ^b^ Percent receptor activation at 100 μM; ^c^ Efficacy (E_max_) relative to the maximal effect of the full agonist decanoic acid (100 μM) (=100%); ^d^ Percent receptor activation at 30 μM; ^e^ Efficacy (E_max_) relative to the maximal effect of the full agonist embelin (10 μM) (= 100%).

## Supplementary Materials

The following are available online at http://www.mdpi.com/1660-3397/16/10/369/s1, Table S1: 16S rDNA Sequence of *Labrenzia* sp. strain 011, Table S2. 1D and 2D NMR spectroscopic data (300 MHz, CDCl_3_) of compound **1**, Table S3. 1D and 2D NMR spectroscopic data (300 MHz, CDCl_3_) of compound **2**, Figure S1: 1H (300 MHz) Spectrum of compound **1** in CDCl_3_, Figure S2: ^13^C (300 MHz) Spectrum of compound **1** in CDCl_3_, Figure S3: DEPT-135 (300 MHz) Spectrum of compound **1** in CDCl_3_, Figure S4: COSY (300 MHz) Spectrum of compound **1** in CDCl_3_, Figure S5: HSQC (300 MHz) Spectrum of compound **1** in CDCl_3_, Figure S6: HMBC (300 MHz) Spectrum of compound **1** in CDCl_3_, Figure S7: 1H (300 MHz) Spectrum of compound **2** in CDCl_3_, Figure S8: ^13^C (300 MHz) Spectrum of compound **2** in CDCl_3_, Figure S9: DEPT-135 (300 MHz) Spectrum of compound **2** in CDCl_3_, Figure S10: COSY (300 MHz) Spectrum of compound **2** in CDCl_3_, Figure S11: HSQC (300 MHz) Spectrum of compound **2** in CDCl_3_, Figure S12: NOESY (300 MHz) Spectrum of compound **2** in CDCl_3_, Figure S13: HMBC (300 MHz) Spectrum of compound **2** in CDCl_3_, Figure S14: EIC and (-) HRMS spectrum of compound **1**, Figure S15: EIC and (-) HRMS spectrum of compound **2**, Figure S16: Concentration-response curve of 2 determined in β-arrestin assays using the β-galactosidase complementation technology, Figure S17: Average amino acid identity (ANI) heat map of the *Labrenzia* strains, Figure S18: CAFS1 gene cluster alignment in *Labrenzia* strains, Figure S19: CAFS2 gene cluster alignment in *Labrenzia* strains, Figure S20: CAFS3 gene cluster alignment in *Labrenzia* strains.
