# Supplementary material for: Cyclopropane-Containing Fatty Acids from the Marine Bacterium Labrenzia sp. 011 with Antimicrobial and GPR84 Activity
Source: Mar Drugs. 2018 Oct 8;16(10):369. doi: 10.3390/md16100369 (PMC6213206; doi:10.3390/md16100369)
Supplement: Supplementary file 1 [file marinedrugs-16-00369-s001.pdf]

Supporting information

Cyclopropane-containing fatty acids from the marine bacterium *Labrenzia* sp. 011 with antimicrobial and GPR84 activity

Jamshid Amiri-Moghaddam[1,†], Antonio Dávila-Céspedes[1,†], Stefan Kehraus[1], Max Crüsemann[1]; Meryem Köse[2], Christa E. Müller[2] and Gabriele Maria König[1,*]

[1] Institute for Pharmaceutical Biology, University of Bonn, Nussallee 6, 53115 Bonn, Germany; jamirimoghaddam@uni-bonn.de; adavila49@gmail.com
[2] Pharmaceutical Institute, Pharmaceutical Chemistry I, An der Immenburg 4, D-53121 Bonn, Germany; mkoese@uni-bonn.de; christa.mueller@uni-bonn.de
† These authors contributed equally to this article.
* Correspondence: g.koenig@uni-bonn.de; Tel.: +49 228 73 3747

Table S1: 16S rDNA Sequence of *Labrenzia* sp. strain 011

>CGCATGCTCCGGCCGCCATGGCCGCGGGATTAAGGAGGTGATCCAGCCCCAGGTTCCCCTAGGGCT
ACCTTGTTACGACTTCACCCCAGTCGCTGAGCCTACCGTGGTCAGCTGCCTCCTTGCGGTTAGCGCACT
GCCTTCGGGTAAACCCAACTCCCATGGTGTGACGGGCGGTGTGTACAAGGCCCGGGAACGTATTCAC
CGCGTCATGCTGTTACGCGATTACTAGCGATTCCAACTTCATGCTCTCGAGTTGCAGAGAACAATCCG
AACTGAGACGGCTTTTGGAGATTAGCTCCCTCTCGCGAGTTCGCTGCCCACTGTCACCGCCATTGTAG
CACGTGTGTAGCCCAGCCCGTAAGGGCCATGAGGACTTGACGTCATCCCCACCTTCCTCTCGGCTTAT
CACCGGCAGTCCCCCTAGAGTGCCCAACTTAATGCTGGCAACTAAGGGCGAGGGTTGCGCTCGTTGC
GGGACTTAACCCAACATCTCACGACACGAGCTGACGACAGCCATGCAGCACCTGTCCTGGCGTCCCC
GAAGGGAACAATCGGTCTCCCGATCTAGCACCAAATGTCAAGGGCTGGTAAGGTTCTGCGCGTTGCT
TCGAATTAAACCACATGCTCCACCGCTTGTGCGGGCCCCCGTCAATTCCTTTGAGTTTTAATCTTGCGA
CCGTACTCCCCAGGCGGGAAGCTTAATGCGTTAGCTGCGCCACCAAATAGCATGCTACCTGACGGCT
AGCTTCCATCGTTTACGGCGTGGACTACCAGGGTATCTAATCCTGTTTGCTCCCCACGCTTTCGCACCT
CAGCGTCAGTACCGAGCCAGTGAGCCGCCTTCGCCACTGGTGTTCTTCCGAATATCTACGAATTTCGC
CTCTACACTCGGAGTTCCACTCACCTCTCTCGGTCTCAAGACTGACAGTATCAAAGGCAGTTCCGGGG
TTGAGCCCCGGGATTTCACCCCTGACTGATCAGTCCGCCTACGTGCGCTTTACGCCCAGTGATTCCGA
ACAACGCTAGCCCCCTTCGTATTACCGCGGCTGCTGGCACGAAGTTAGCCGGGGCTTCTTCTGCGAGT
AACGTCATTATCCTCCTCGCTGAAAGAGCTTTACAACCCTAGGGCCTTCATCACTCACGCGGCATGGC
TGGATCAGGGTTGCCCCCATTGTCCAATATTCCCCACTGCTGCCTCCCGTAGGAGTCTGGGCCGTGTCT
CAGTCCCAGTGTGGCTGATCATCCTCTCAGACCAGCTATGGATCGTCGCCTTGGTAGGCCATTACCCC
ACCAACTAGCTAATCCAACGCGGGCCCATCCTTAGGCGATAAATCTTCCCCCCATAGGGCACATACG
GTATTAGCAGTCGTTTCCAACTGTTGTTCCGTACCTAAAGGTAGGTTCCCACGCGTTACTCACCCGTCT
GCCACTAACTCCGAAGAGTTCGTTCGACTTGCATGTGTTAAGCCTGCCGCCAGCGTTCGTTCTGAGCC
AGGATCAAACTCTAATCACTAGTGCGGCCGCCTGCAGGTCGACCATATGGGAGAGCTCCCAACGCGT
GA

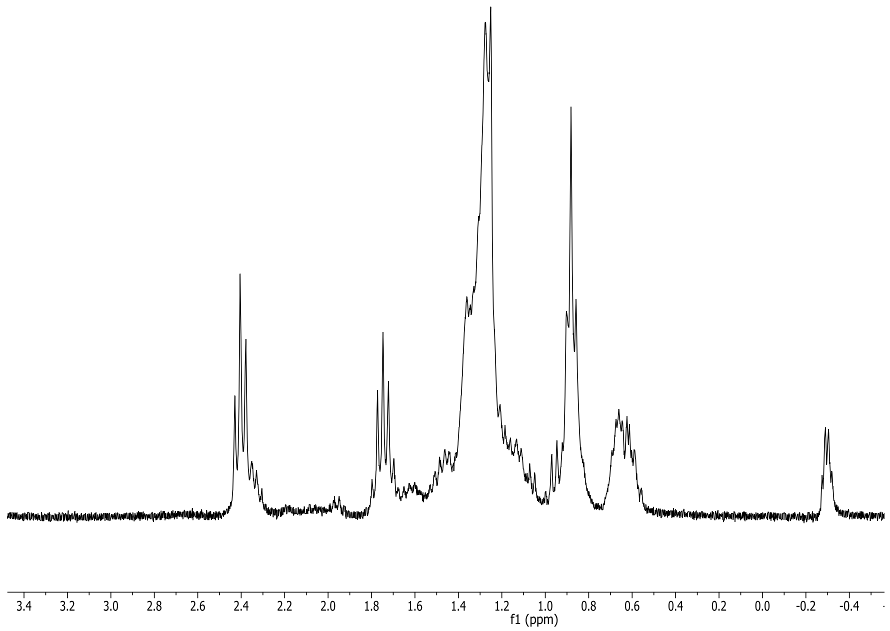

Figure S1: ^{1}H (300 MHz) Spectrum of compound **1** in $CDCl_3$

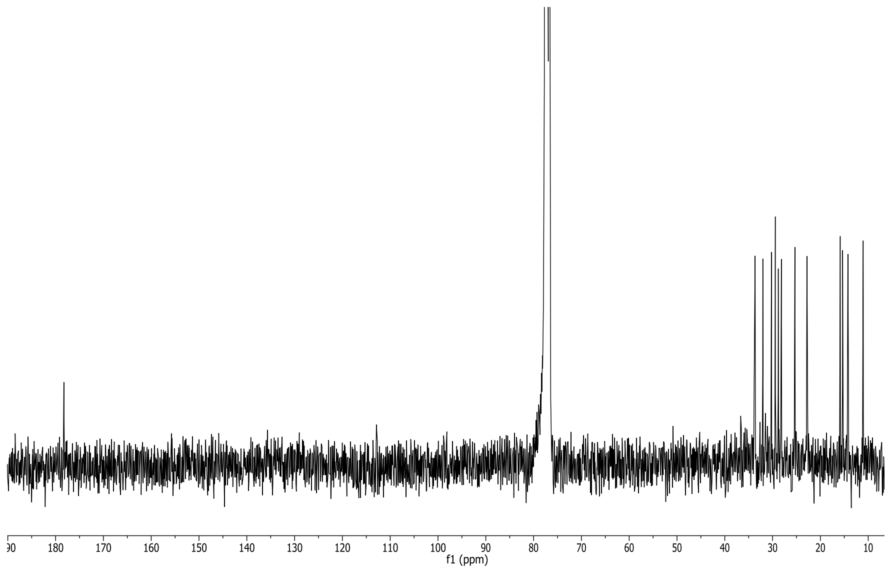

Figure S2: ^{13}C (300 MHz) Spectrum of compound **1** in $CDCl_3$

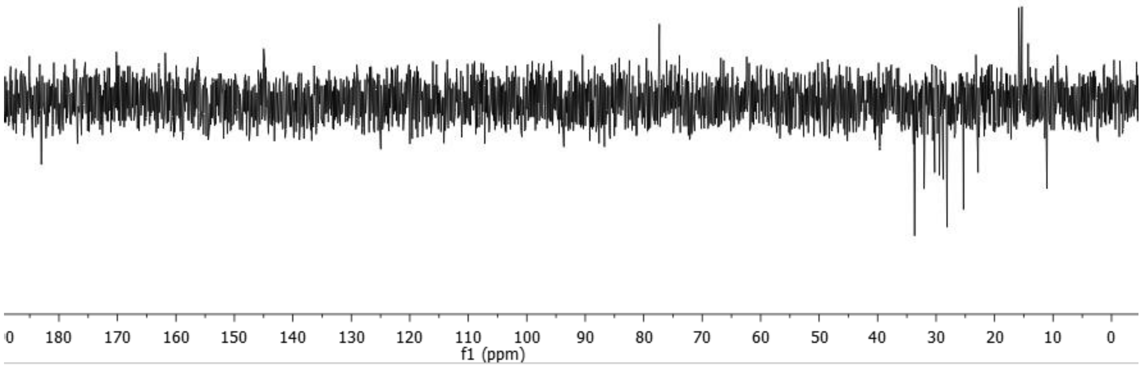

Figure S3: DEPT-135 (300 MHz) Spectrum of compound **1** in $CDCl_3$

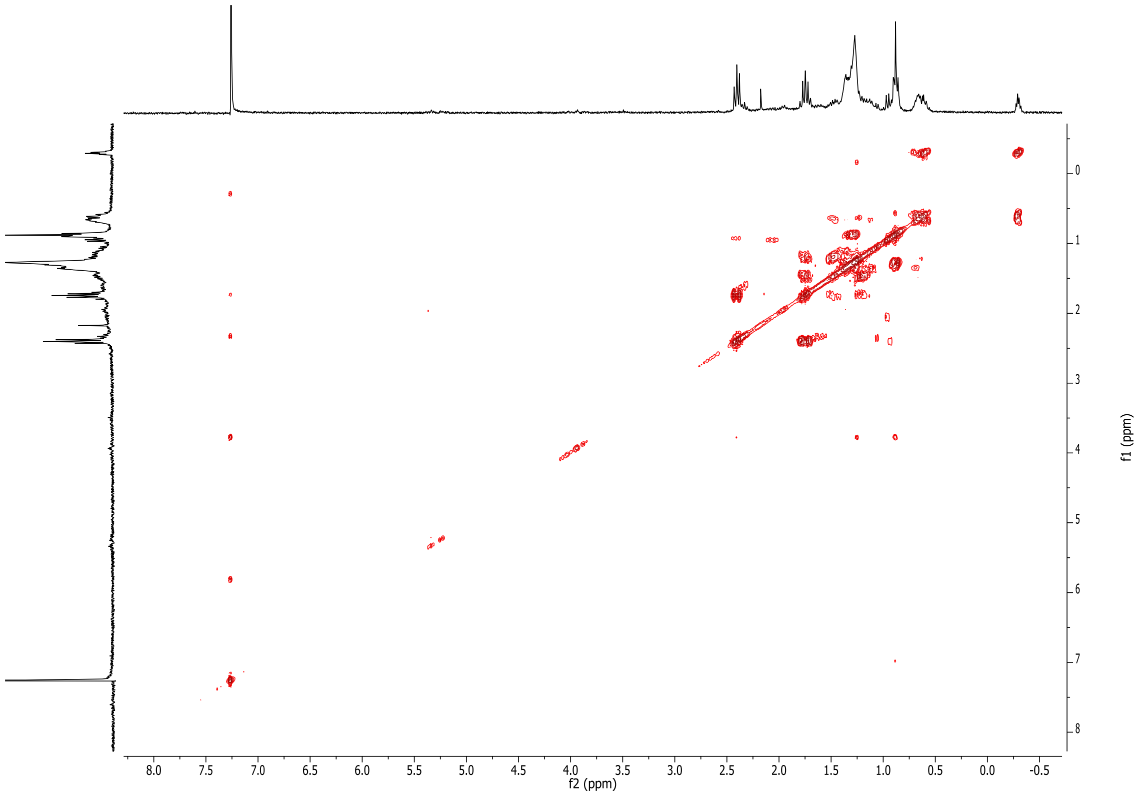

Figure S4: COSY (300 MHz) Spectrum of compound **1** in $CDCl_3$

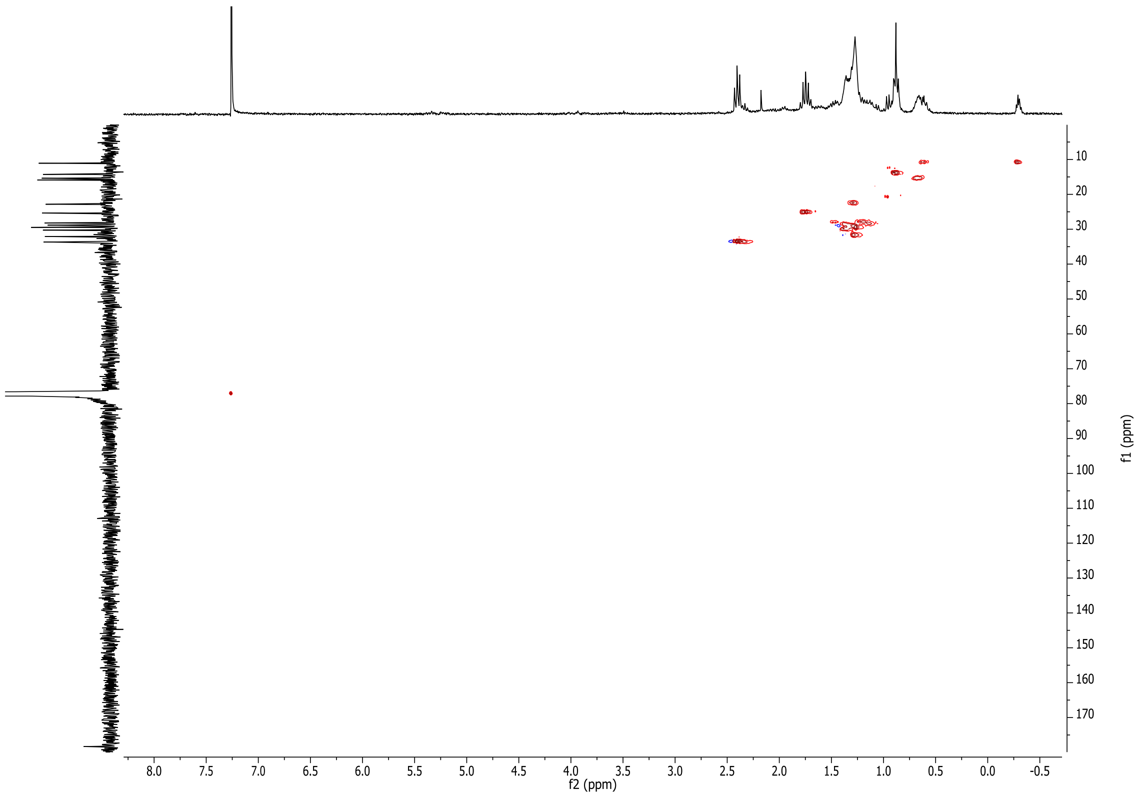

Figure S5: HSQC (300 MHz) Spectrum of compound **1** in $CDCl_3$

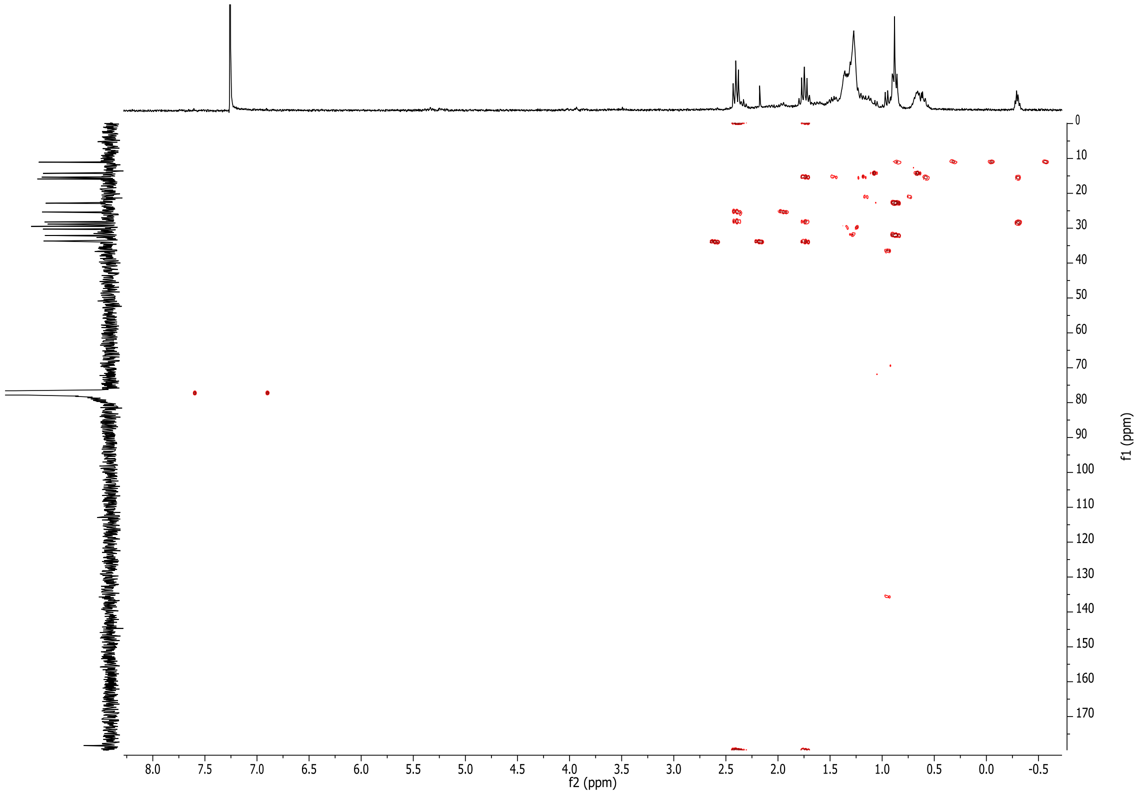

Figure S6: HMBC (300 MHz) Spectrum of compound **1** in $CDCl_3$

Table S2. 1D and 2D NMR spectroscopic data (300 MHz, $CDCl_3$) of compound **1**

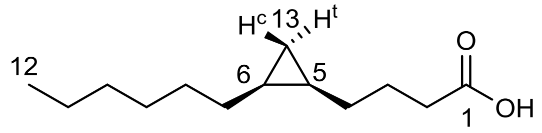

Pos.	δ_C, mult [ppm]	δ_H (*J* in Hz) [ppm]	COSY	HMBC
1	178.1, C	-	-	-
2	33.5, CH_2	2.40, t (7.6)	3	1, 3, 4
3	25.2, CH_2	1.75, p (7.6)	2, 4	1, 2, 4, 5
4	28.0, CH_2	a:1.46, m<br>b: 1.22, m	3, 4b, 5<br>3, 4a, 5	5<br>5
5	15.2, CH	0.66, m	4a/b, 13a/b	-
6	15.7, CH	0.68, m	7	-
7	28.7, CH_2	1.35, m	-	-
8	29.3, CH_2	1.25, m	-	-
9	30.1, CH_2	1.35, m	-	-
10	31.9, CH_2	1.27, m	-	-
11	22.7, CH_2	1.28, m	12	-
12	14.1, CH_3	0.88, t (6.8)	11	10, 11
13	10.9, CH_2	a^t: 0.63, m<br>b^c: -0.30, q (4.6)	5, 6, 13b<br>5, 6, 13a	4, 5, 6, 7<br>4, 5, 6, 7

^{c}*cis*-configured proton; ^{t}*trans*-configured proton

Table S3. 1D and 2D NMR spectroscopic data (300 MHz, $CDCl_3$) of compound **2**.

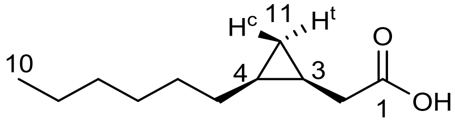

Pos.	δ_C, mult [ppm]	δ_H (*J* in Hz) [ppm]	COSY	HMBC	NOESY
1	180.2, C	-	-	-	-
2	33.7, CH_2	a: 2.42, dd (6.9, 16.0)<br>b: 2.29, dd (7.8, 16.0)	2b, 3<br>2a, 3	1, 3, 4, 11<br>1, 3, 4, 11	2b, 3<br>2a, 3
3	11.1, CH	1.10, m	2, 4, 11a/b	-	11a
4	15.5, CH	0.81, m	3, 11a/b	-	-
5	29.8, CH_2	1.37, m	-	-	-
6	28.8, CH_2	1.35, m	-	-	-
7	29.2, CH_2	1.29, m	-	-	-
8	31.9, CH_2	1.26, m	-	-	-
9	22.6, CH_2	1.29, m	10	-	10
10	14.1, CH_3	0.88, t (7.3)	9	8, 9	9
11	10.8, CH_2	a^t: 0.75, dq (8.4, 5.0)<br>b^c: -0.13, q (5.0)	3, 4, 11b<br>3, 4, 11a	2, 3, 4, 5<br>2, 3, 4, 5	3, 11b<br>11a

^{c}*cis*-configured proton; ^{t}*trans*-configured proton

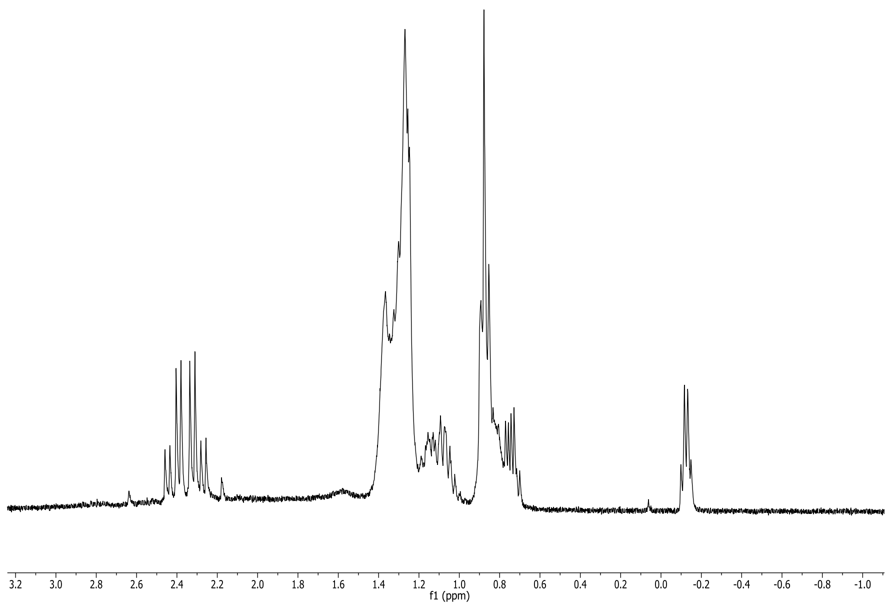

Figure S7: ^{1}H (300 MHz) Spectrum of compound **2** in $CDCl_3$

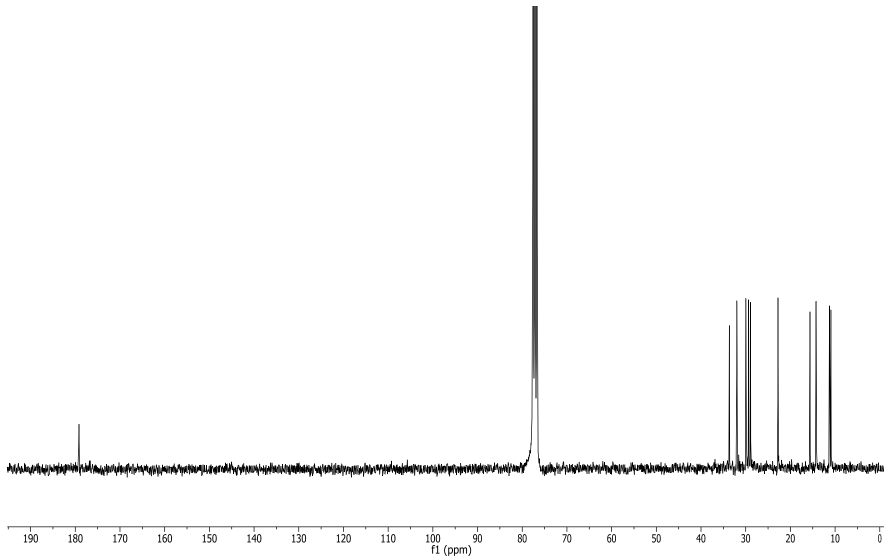

Figure S8: ^{13}C (300 MHz) Spectrum of compound **2** in $CDCl_3$

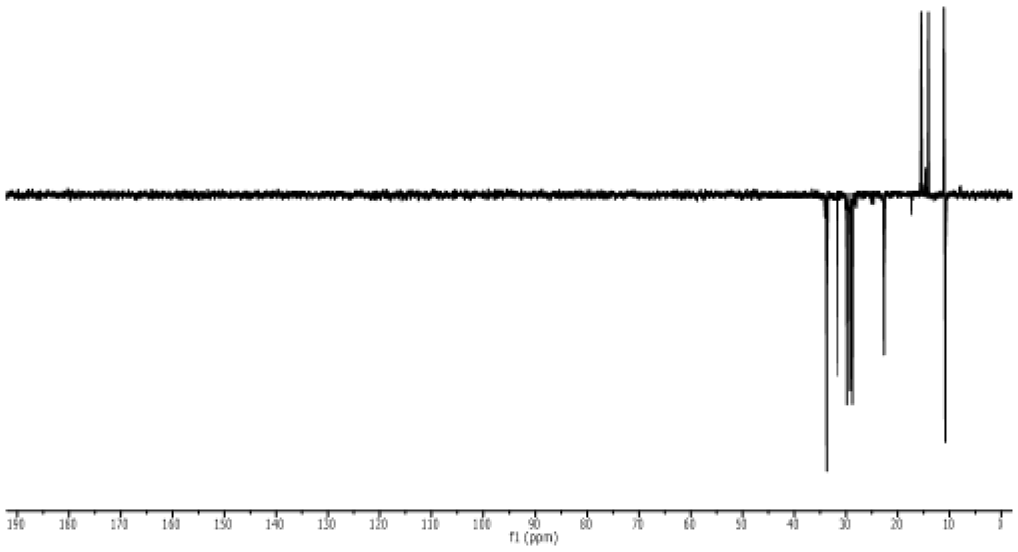

Figure S9: DEPT-135 (300 MHz) Spectrum of compound **2** in $CDCl_3$

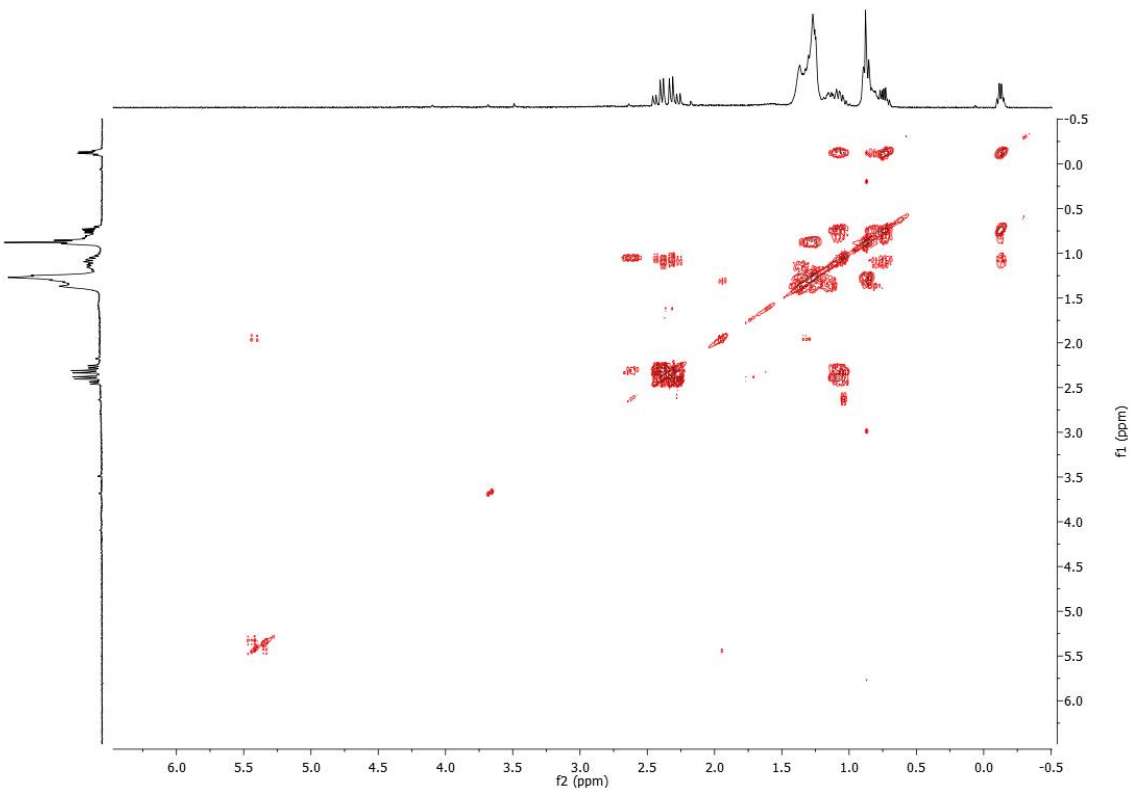

Figure S10: COSY (300 MHz) Spectrum of compound **2** in $CDCl_3$

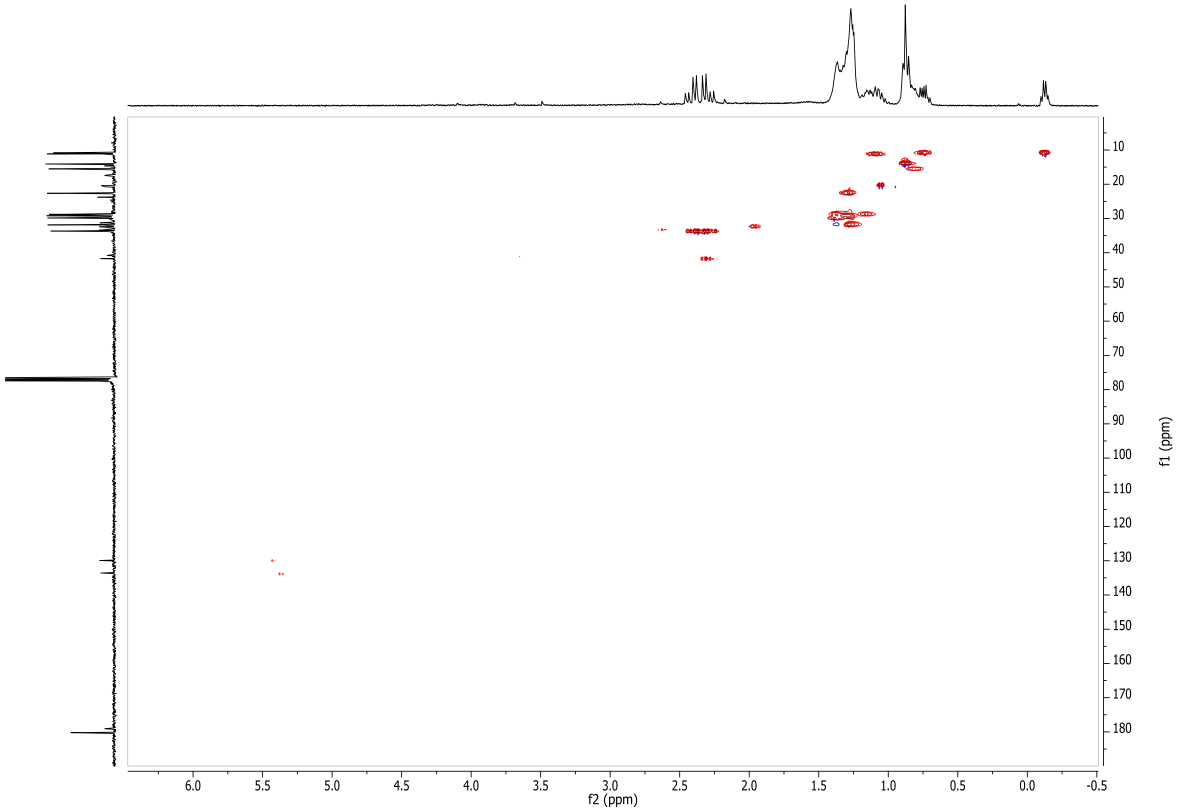

Figure S11: HSQC (300 MHz) Spectrum of compound **2** in $CDCl_3$

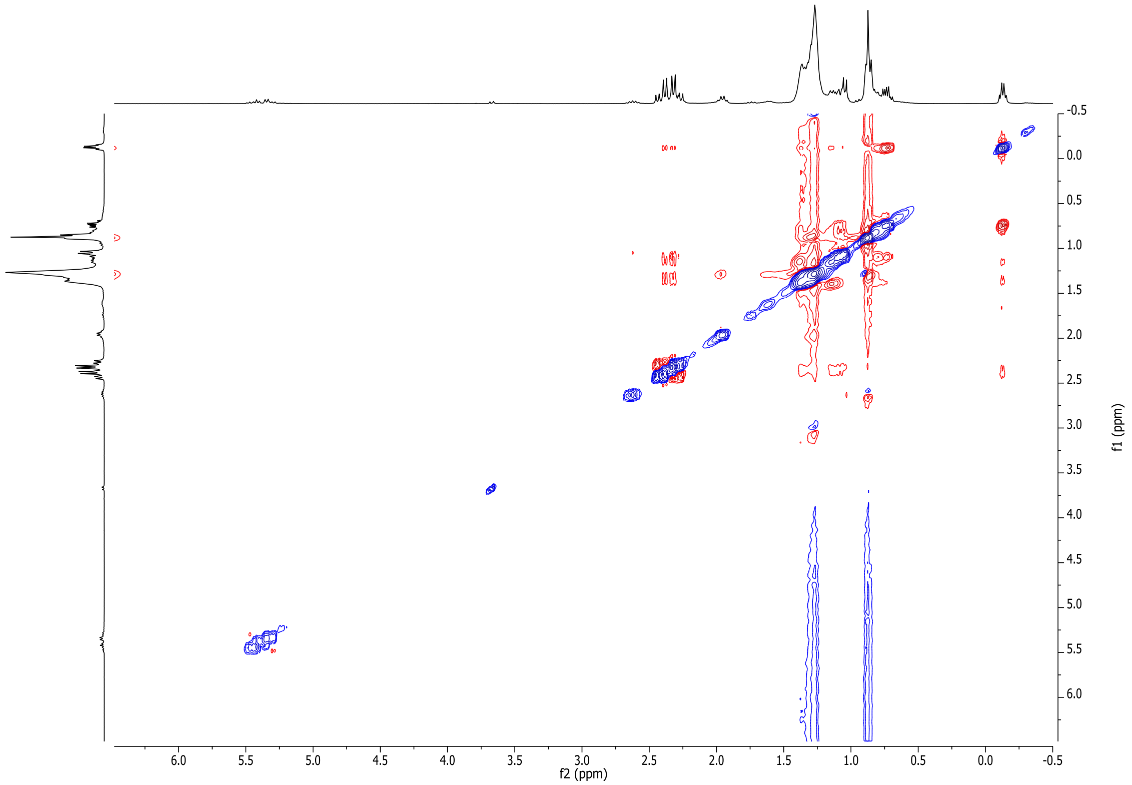

Figure S12: NOESY (300 MHz) Spectrum of compound **2** in $CDCl_3$

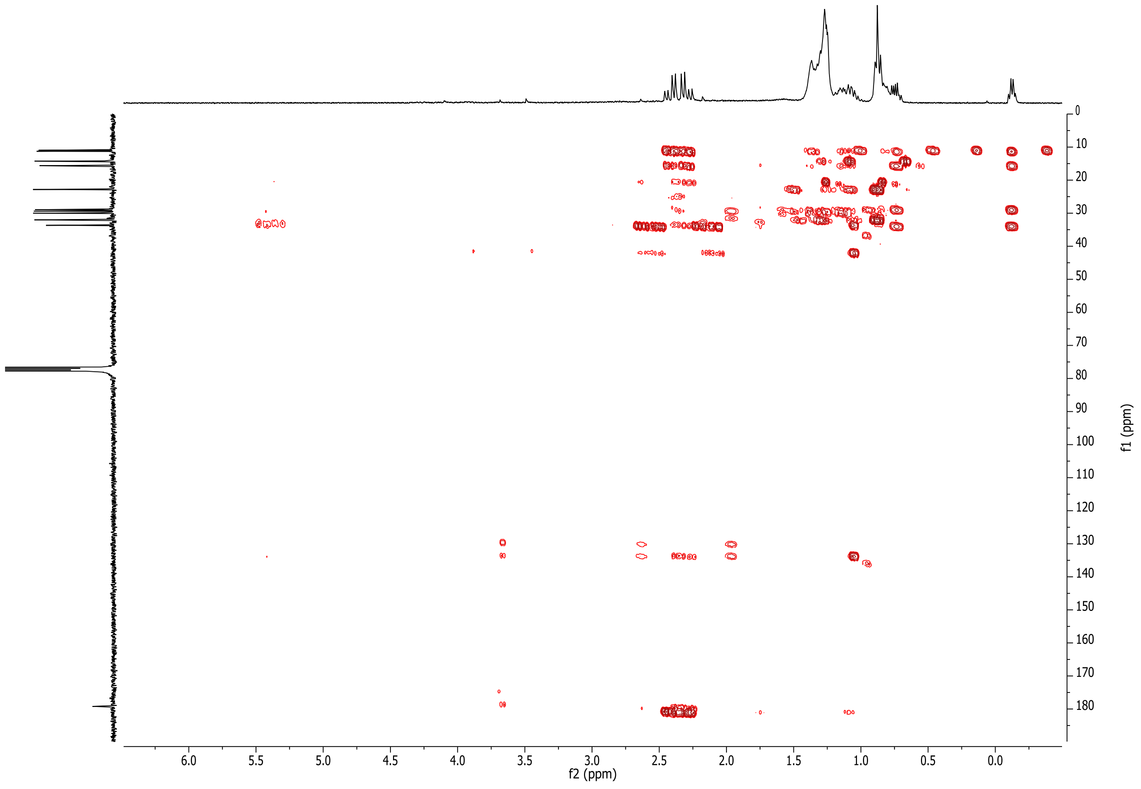

Figure S13: HMBC (300 MHz) Spectrum of compound **2** in $CDCl_3$

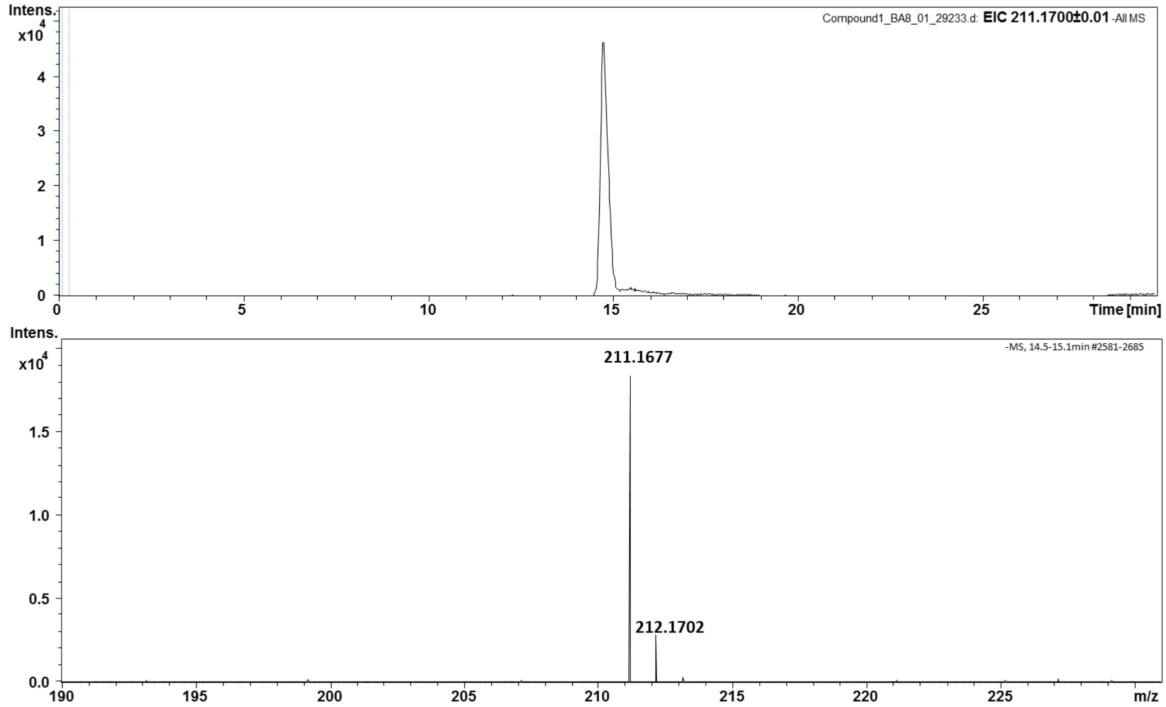

Figure S14: EIC and (-) HRMS spectrum of compound **1**, calculated *m/z* for $C_{13}H_{23}O_2$: 211.1698 / Measured: 211.1677)

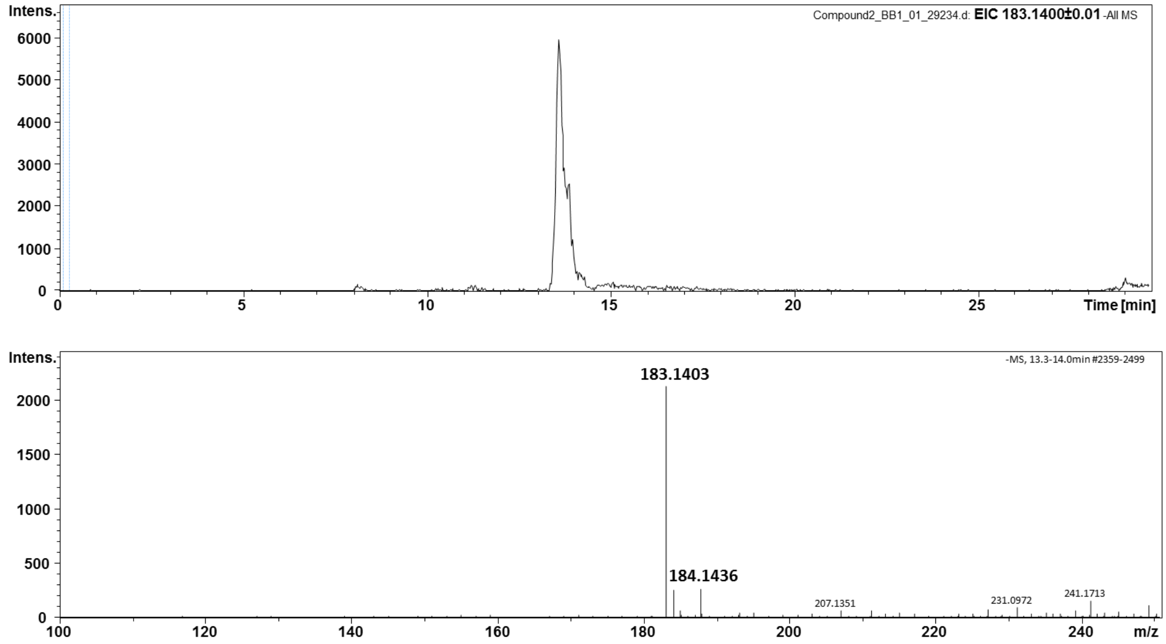

Figure S15: EIC and (-) HRMS spectrum of compound **2**, calculated *m/z* for $C_{11}H_{29}O_2$: 183.1385 / Measured: 183.1403)

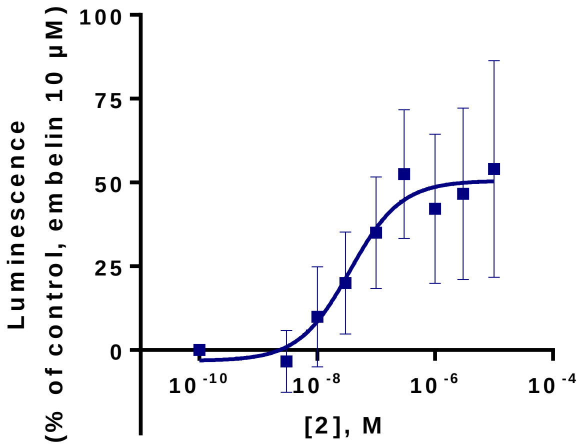

Figure S16. Concentration-response curve of **2** determined in β-arrestin assays using the β-galactosidase complementation technology. The maximal luminescence induced by the full agonist embelin (10 µM) was defined as 100 %. The buffer control was defined as 0 %. An EC_{50} value of 114 ± 135 nM was calculated for **2**. Mean values ± SD from 5 independent experiments performed in duplicates are shown.

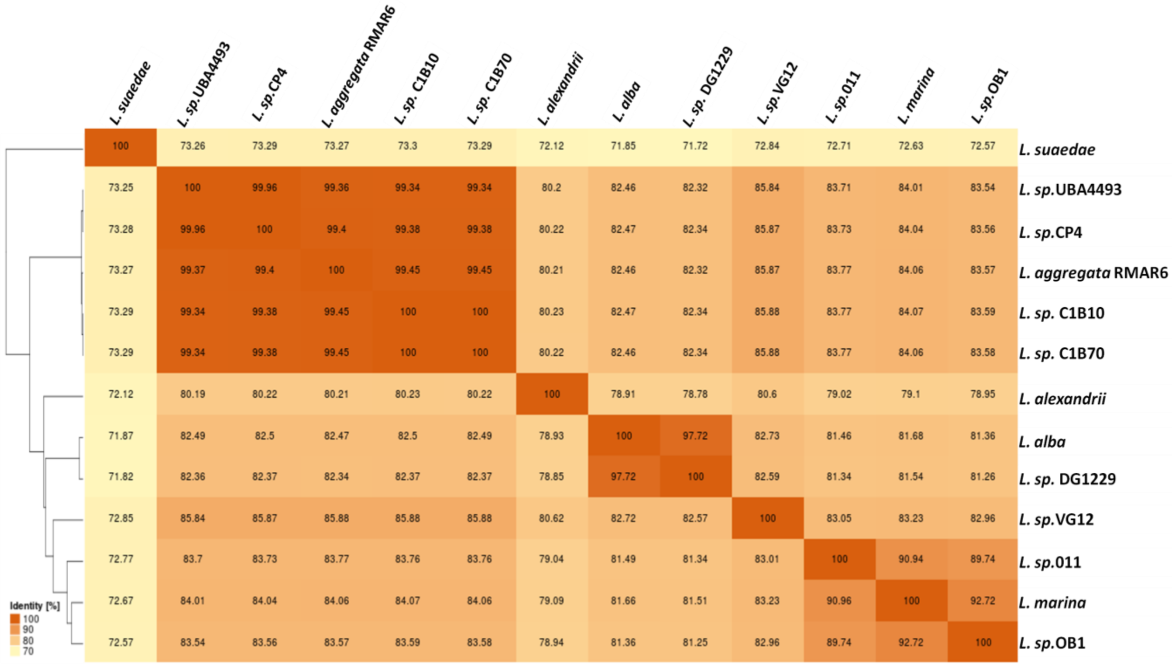

Figure S17. Average amino acid identity (ANI) heat map of the *Labrenzia* strains.

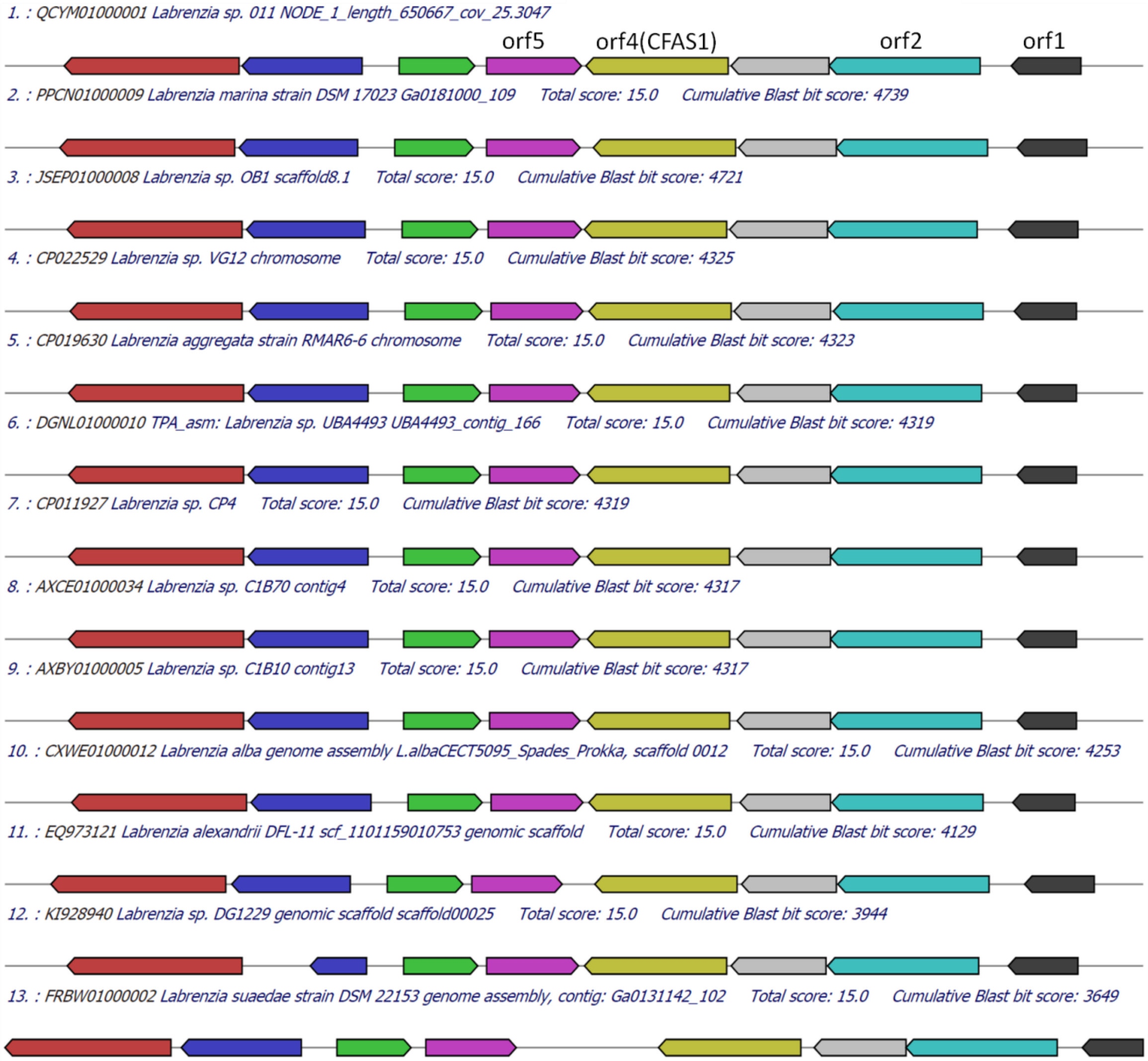

Figure S18. CAFS1 gene cluster alignment in *Labrenzia* strains, orf2: FAD-dependent oxidoreductase, orf4: Cyclopropane fatty acyl-phospholipid synthase (CFAS1), and orf5: short-chain dehydrogenase/reductase SDR. Same colors represent the same annotation.

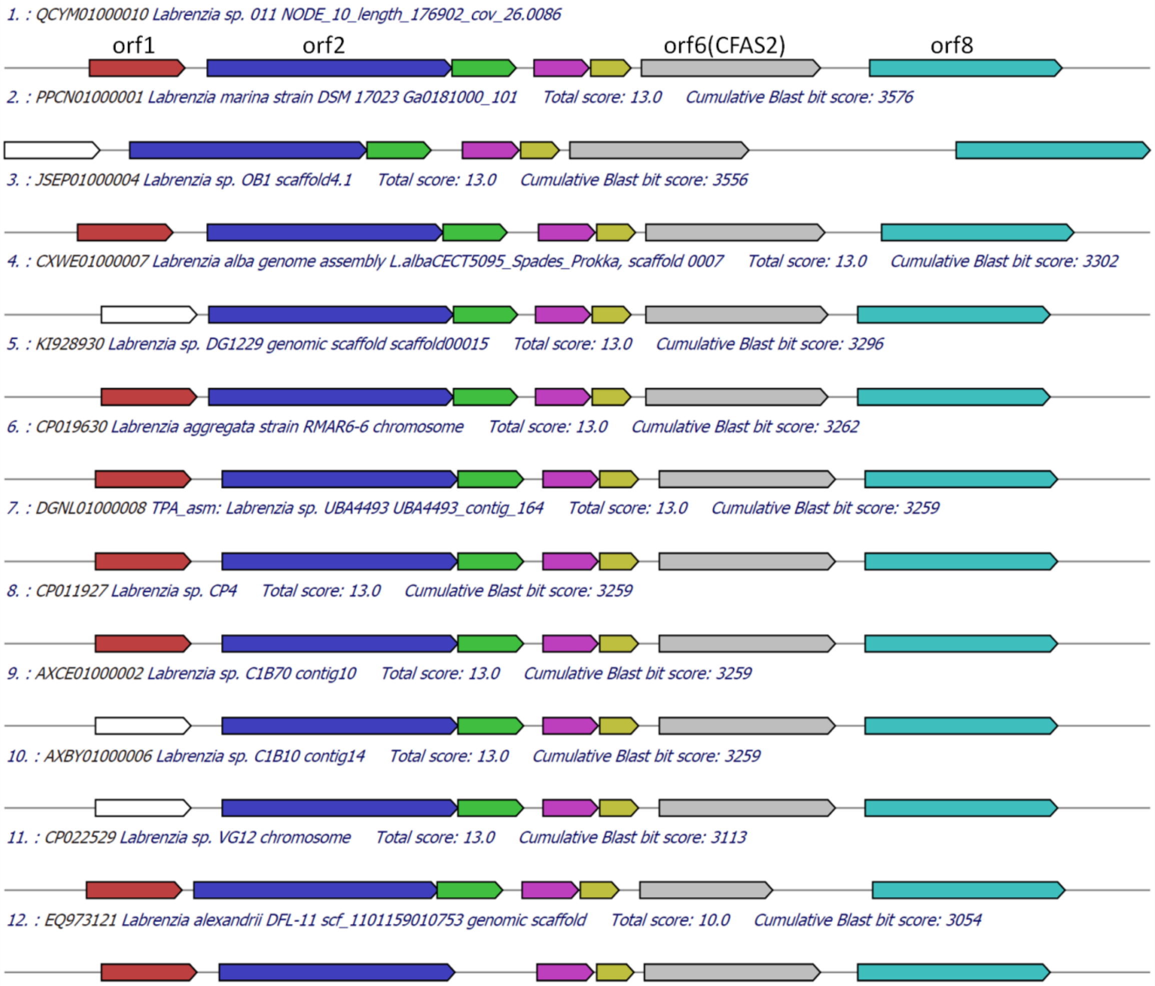

Figure S19. CAFS2 gene cluster alignment in *Labrenzia* strains, orf2: Sensor histidine kinase, orf4: Cyclopropane fatty acyl-phospholipid synthase (CFAS2), and orf8: Adenosylhomocysteinase. Same colors represent the same annotation.

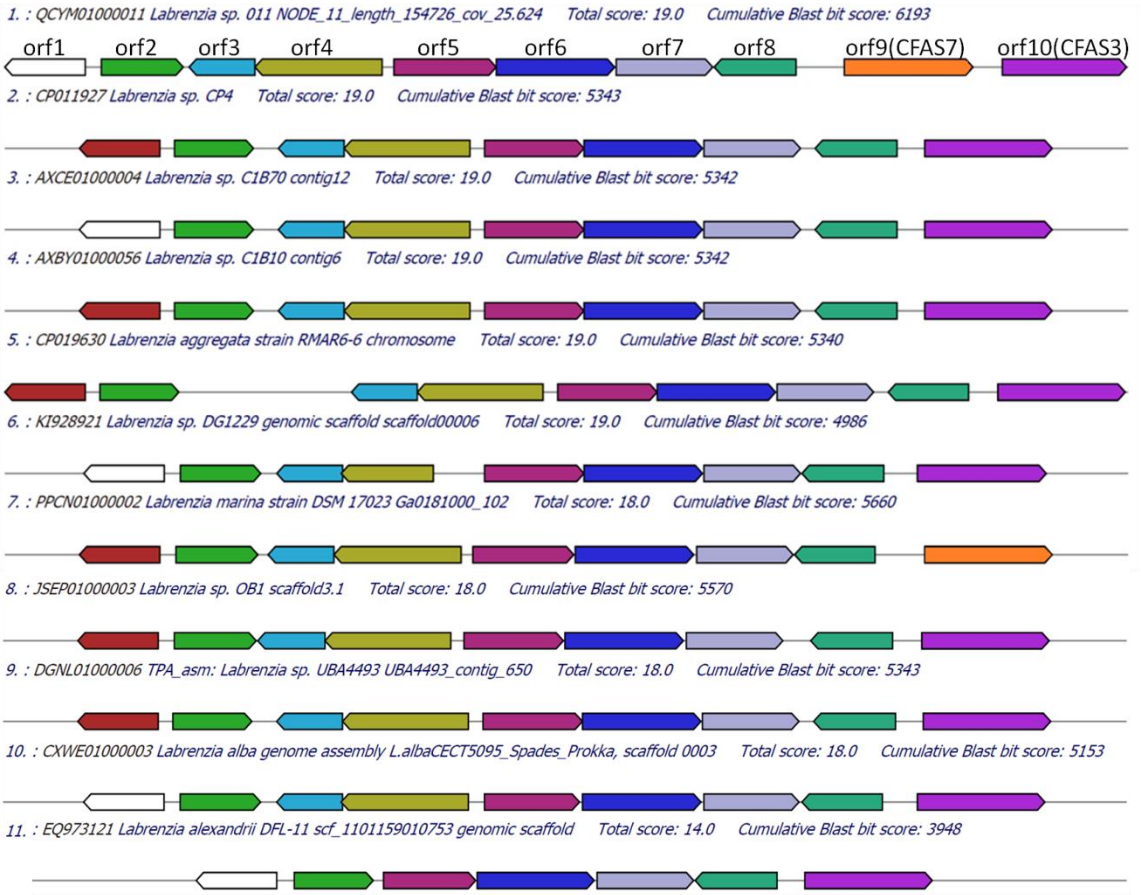

Figure S20. CAFS3 gene cluster alignment in *Labrenzia* strains, orf1: metallo-beta-lactamase, orf2: methyltransferase, orf3: methyltransferase, orf4: homoserine_O-acetyltransferase, orf5: Chorismate mutase, orf6: aminotransferase, orf7: 3-hydroxyisobutyrate dehydrogenase, orf8: Polyprenyl synthetase, orf9: Cyclopropane fatty acyl-phospholipid synthase (CFAS7), and orf10: Cyclopropane fatty acyl-phospholipid synthase (CFAS3). Same colors represent the same annotation.

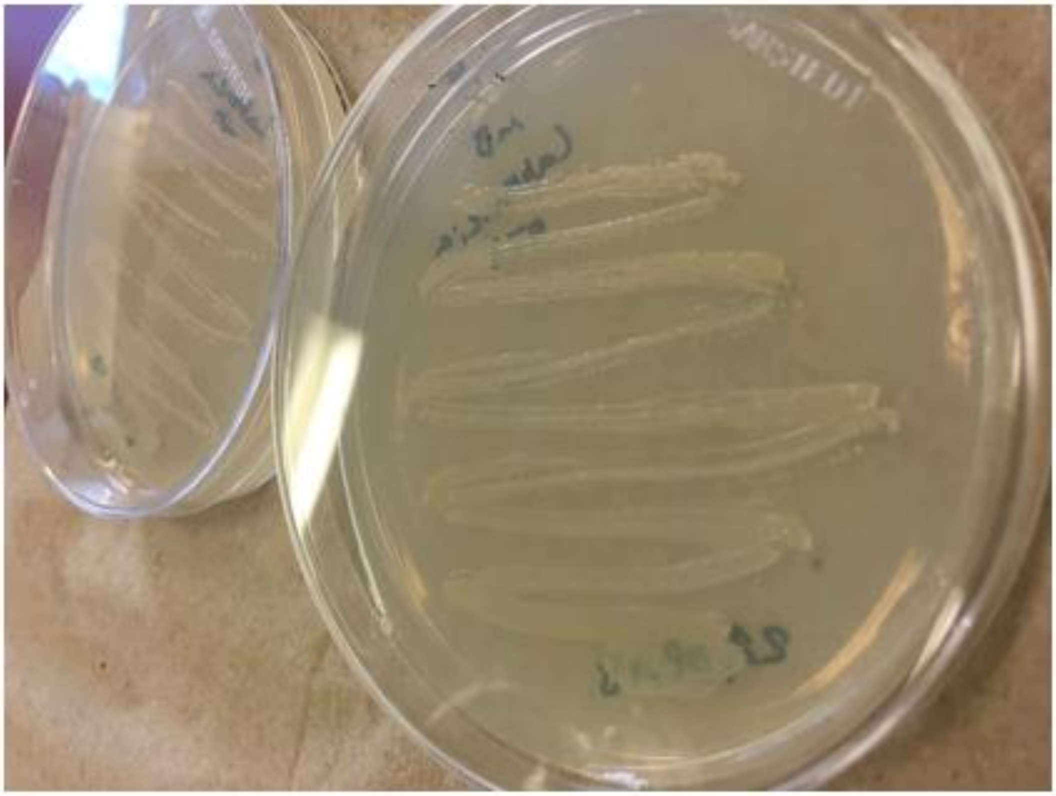

Figure S21. Colonies of *Labrenzia* sp. 011 on marine agar (Difco 2216).
